# TTL-Expression Modulates Epithelial Morphogenesis

**DOI:** 10.3389/fcell.2021.635723

**Published:** 2021-02-05

**Authors:** Manuel Müller, Karina Ringer, Florian Hub, Natalia Kamm, Thomas Worzfeld, Ralf Jacob

**Affiliations:** ^1^Department of Cell Biology and Cell Pathology, Philipps-Universität Marburg, Marburg, Germany; ^2^DFG Research Training Group, Membrane Plasticity in Tissue Development and Remodelling, Philipps-Universität Marburg, Marburg, Germany; ^3^Institute of Pharmacology, Biochemical-Pharmacological Center, University of Marburg, Marburg, Germany; ^4^Max-Planck-Institute for Heart and Lung Research, Department of Pharmacology, Bad Nauheim, Germany

**Keywords:** tubulin tyrosine ligase, microtubule tyrosination/detyrosination, intestinal organoid, focal adhesion, epithelia cells

## Abstract

Epithelial monolayer formation depends on the architecture and composition of the microtubule cytoskeleton. Microtubules control bidirectional trafficking and determine the positioning of structural cellular proteins. We studied the role of tubulin tyrosination in epithelial cell shape and motility. Tubulin tyrosine ligase (TTL), the enzyme that adds tyrosine to the carboxy terminus of detyrosinated α-tubulin, was depleted or overexpressed in 2D epithelial monolayers as well as in 3D intestinal organoids. We demonstrate qualitatively and quantitatively that in the absence of TTL the cells comprise high levels of detyrosinated tubulin, change their shape into an initial flat morphology and retardedly acquire a differentiated columnar epithelial cell shape. Enhanced adhesion and accelerated migration patterns of TTL-knockout cells combined with reverse effects in TTL-overexpressing cells indicate that the loss of TTL affects the organization of cell adhesion foci. Precipitation of detyrosinated tubulin with focal adhesion scaffold components coincides with increased quantities and persistence of focal adhesion plaques. Our results indicate that the equilibrium between microtubules enriched in detyrosinated or tyrosinated tubulin modulates epithelial tissue formation, cell morphology, and adhesion.

## Introduction

Dynamic arrangement and structured architecture of cytoskeletal elements ensure formation and maintenance of epithelial cell sheets. Microtubules can be modulated by posttranslational modifications, which include acetylation, tyrosination, detyrosination, Δ2 modification, polyglutamylation, palmitoylation and phosphorylation (Janke and Magiera, 2020; Roll-Mecak, 2020). Tyrosinated (tyr-tubulin), and detyrosinated (detyr-tubulin) α-tubulin is generated by a cycle of removal and subsequent religation of tyrosine to the carboxy terminus of this polypeptide. Detyrosination can inhibit microtubule disassembly, whereas dynamic microtubules are predominantly tyrosinated (Palazzo et al., 2004). Very recently, the vasohibins VASH1 and its homolog VASH2 have been identified to remove the C-terminal tyrosine of α-tubulin (Aillaud et al., 2017; Nieuwenhuis et al., 2017). The tubulin tyrosination cycle is then completed by the enzyme tubulin tyrosine ligase (TTL), which catalyzes C-terminal α-tubulin tyrosination on αβtubulin heterodimers and restores tyr-tubulin (Raybin and Flavin, 1975). Length, amino acid composition and additional modifications of the C-terminal α-tubulin tail have most likely no influence on α-tubulin tyrosination by TTL (Prota et al., 2013). In myocytes shRNA-mediated TTL-depletion increased detyrosination, cell viscosity and contractile resistance, thus altering myocyte mechanics (Robison et al., 2016).

Expression of TTL in general affects cell fate and there is a widespread loss of TTL activity during tumor growth *in situ* (Lafanechere et al., 1998). In accordance, an increased level of detyr-tubulin in breast tumors predicts poor patient survival and an enhanced risk of cancer-related complications (Mialhe et al., 2001). Turnover of adhesive structures at the front of migrating cells can be controlled by intracellular traffic along microtubules for polarized delivery of adhesion receptors, such as integrins (Bretscher and Aguado-Velasco, 1998). Microtubules thus regulate migration speed (Stehbens and Wittmann, 2012) and their growth provides forces for advancement of the cell edge (Balzer et al., 2012). Recent evidence suggests that microtubule acetylation promotes fast focal adhesion turnover rates and cell migration velocity (Bance et al., 2019). Moreover, in detached mammary epithelial cell lines detyrosinated microtubules are enriched in long and dynamic protrusions of the plasma membrane (Whipple et al., 2007), which facilitates reattachment and suggests that cell adhesion is immediately linked to the microtubule architecture. Mechanistic features of this link and how it can be translated into physiological 3D tissue environments is not clarified yet.

This prompted us to examine the morphology and adhesion of epithelial cells in 2D cell culture as well as in 3D intestinal organoids, in which the α-tubulin tyrosinating enzyme TTL has been overexpressed or knocked out. In the absence of TTL adherent cells in culture or forming organoids dramatically increase the number of detyrosinated tubules. The cells have a flat spread morphology and retardedly differentiate into columnar epithelial monolayers. These morphological alterations following depletion of TTL are further reflected in intestinal organoid epithelia and enterocytes of the small intestine. Cultured cells adhere stronger and migrate faster if TTL is knocked out. Reverse effects in TTL-overexpressing Caco-2 or Madin-Darby Canine Kidney (MDCK) cells indicate that the loss of TTL affects the organization of cell adhesion foci. The knockout of TTL seems to affect focal adhesion dynamics and stability as evidenced by diminished recycling of integrin adhesion receptors, variable pulldown efficiencies of vital focal adhesion components and a longer persistence of vinculin at cell adhesion foci.

## Materials and Methods

### Antibodies and DNA Constructs

The following tubulin antibodies were used: monoclonal anti-α-tubulin (Clone DM 1A) and anti-acetylated α-tubulin (Clone 6-11B-1) (Sigma-Aldrich), monoclonal anti-tyrosinated α-tubulin (YL1/2, Santa Cruz), and polyclonal anti-detyrosinated α-tubulin (Millipore). The following polyclonal antibodies were used: anti-GAPDH (HyTest), anti-Kif5A (Abcam), and anti-TTL (Proteintech Group). The following monoclonal antibodies were used: anti-β-catenin (Sigma-Aldrich), anti-KANK1 (Invitrogen), anti-paxillin (BD Transduction Laboratories), anti-sc35 (Abcam), and anti-vinculin (Sigma-Aldrich). The monoclonal antibody directed against sucrase-isomaltase (SI) (DRBB2/158) was generously provided by A. Quaroni. The plasmid mCherry-Vinculin-N-21 was a gift from Michael Davidson (Addgene plasmid #55160; RRID:Addgene_55160).

### Cell Culture and Transfections

Madin-Darby Canine Kidney type II and MDCK_Δ__*TTL*_ cells were cultured at 37°C under 5% CO_2_ in minimum essential medium (MEM; Gibco) supplemented with 5% fetal calf serum (FCS), 2 mM glutamine, 100 U/ml penicillin, and 100 mg/ml streptomycin. MEM medium for MDCK_*TTL–GFP*_ cells contained 0.5 mg/ml G418 additionally. For the generation of MDCK_Δ__*TTL*_ cells, TTL expression was eliminated by CRISPR/Cas9 gene editing as described below. Plasmid transfection of MDCK cells was performed with Lipofectamine 2000 (Invitrogen) according to the manufacturer’s instructions.

### CRISPR/Cas9 Gene Editing

The plasmid pSpCas9n(BB)-2A-Puro (PX462) V2.0 was a gift from Feng Zhang (Addgene plasmid # 62987). Oligo pairs encoding the 20-nt guide sequences against canine TTL (5′-CAC CGA ATA TCT ACC TCT ATA AAG A-3′, 5′-AAA CTC TTT ATA GAG GTA GAT ATT C-3′) were annealed and ligated into the *Bbs*I digested plasmid to generate pCRISPR-Cas9 ΔTTL (Ran et al., 2013). Following transfection of pCRISPR-Cas9 ΔTTL, cells were selected for 48 h with 2 μg/ml puromycin (Sigma-Aldrich). Single clones were transferred to 12 well plates with Trypsin/EDTA-soaked Whatman slices. Lysates of MDCK cell clones were analyzed for the presence of TTL by immunoblot with pAb anti-TTL antibody. Only those clones were selected that showed no TTL expression.

### Cloning of Lentiviral shRNA Plasmids

The lentiviral inducible shRNA expression plasmids were cloned as follows: targeting sequences against murine TTL were selected from the database provided by the RNAi Consortium and a forward and reverse single-strand oligonucleotide strand was designed containing the required shRNA sequence and *Eco*RI/*Xho*I overhangs for cloning (mTTL_sh2_fwd: 5′-TCG AGA AGG TAT ATT GCT GTT GAC AGT GAG CGC TCC AGA GGA AAG AGA GAG AAT AGT GAA GCCA CAG ATG TAT TCT CTC TCT TTC CTC TGG AGT GCC TAC TGC CTC GG-3′; mTTL_sh2_rev: 5′-AAT TCC GAG GCA GTA GGC ACT CCA GAG GAA AGA GAG AGA ATA CAT CTG TGG CTT CAC TAT TCT CTC TCT TTC CTC TGG AGC GCT CAC TGT CAA CAG CAA TAT ACC TTC-3′). The oligonucleotides were mixed at a ratio of 1:1 in annealing buffer (100 mM NaCl, 10 mM Tris) to a final concentration of 4.5 μM, heated to 95°C in a water bath and left to cool to room temperature in the water. The mix was diluted by 1:400 in 0.5× annealing buffer. In parallel, the target plasmid LT3GEPIR was linearized by restriction digest with *Xho*I and *Eco*RI, followed by ligation with the annealed oligonucleotide (Fellmann et al., 2013).

### Protein Analysis Procedures, Lysate Preparation and Immunoblotting

For preparation of cell lysates the cells were washed with sterile filtered PBS^++^ (PBS supplemented with 1 mM MgCl_2_ and 1 mM CaCl_2_), collected in lysis buffer (150 mM Tris, pH 8; 150 mM NaCl, 150 mM EDTA, 1% Triton X-100, freshly added protease inhibitor cocktail) after the indicated time intervals and incubated at 4°C on a rotating platform for 30 min. Afterward samples were centrifuged for 15 min at 17,000 *g*. The protein concentrations in the supernatants were determined by Lowry and equal protein amounts were separated by SDS-PAGE using the Hoefer-Mini-VE system (Amersham Pharmacia Biotech) and transferred to nitrocellulose membranes. Membranes were blocked in 5% skimmed milk powder in PBS for 1 h and incubated with primary antibodies overnight at 4°C. Detection was performed with horseradish-peroxidase-conjugated secondary antibodies and ECL reagent (Thermo Fischer Scientific) on an Intas gel imager. The results were quantified using LabImage 1D software (see below).

### Immunoprecipitation

Madin-Darby Canine Kidney cells were washed with PBS^++^, collected in PHEM lysis buffer (50 mM PIPES, 50 mM HEPES, 1 mM EDTA, 2 mM MgCl_2_, pH 6.9/2 M glycerol/2% Triton X-100/freshly added protease inhibitor cocktail) by mechanical detachment and incubated at 4°C for 30 min on a rotating platform. After centrifugation (17,000*g* for 15 min), cleared lysates were precleared and incubated with RFP-nanobody agarose (RFP Trap, Chromotek) or anti-vinculin antibodies/protein A-agarose beads for 2 h at 4°C. Blocked protein A-agarose beads (Chromotek) or non-specific IgG/protein A-agarose beads were used as negative control. Finally, beads were rinsed three times with PHEM washing buffer (50 mM PIPES, 50 mM HEPES, 1 mM EDTA, 2 mM MgCl_2_, pH 6.9), once with PBS and boiled in SDS/PAGE loading buffer for western blot analysis.

### Immunofluorescence, Immunostaining of Tissue Samples, Fluorescence Microscopy, and Photoconversion

For immunofluorescence analysis, cells were grown on cover slips or 24-well filter inserts and fixed with 4% paraformaldehyde for 20 min. Afterward, cells were permeabilized with 0.1 or 0.2% Triton-X-100 for 20 min and blocked in 5% BSA/PBS^++^ for 1 h. Immunostaining was performed with the indicated primary antibodies in blocking reagent for 2 h or overnight. Secondary antibodies labeled with the indicated Alexa Fluor dyes were applied in PBS^++^ for 1 h. Nuclei were stained with Hoechst 33342. Following incubation, cells were washed with PBS^++^ and mounted with Mowiol. Intestinal tissue was taken from patients in the Department of Urology and Pediatric Urology, University Medical Center Marburg for diagnostic purpose. The study was positively evaluated by the local ethic commission. The patients were not followed clinically in this study. Four micrometer thick slices of formalin fixed and paraffin embedded human small intestinal samples were steamed in Tris/EDTA for 20 min or in Citrate buffer for 5 min and blocked in 5% goat serum/PBS. Primary and secondary antibodies were incubated in antibody diluent (Dako). Confocal images were acquired on a Leica TCS SP2 microscope equipped with a 40× or 63× oil plan-apochromat objective (Leica Microsystems). For photoconversion experiments widefield microscopy photobleaching experiments were conducted with N-terminal fusions of vinculin to mEOS2 (Stubb et al., 2019). Transfected MDCK cells were imaged in a 37°C incubation chamber using a 40× oil immersion objective on a Leica DMI8 microscope and photoconversion was accomplished using the Leica infinity scanner module with a 405 nm laser for localized mEOS2-conversion. Epifluorescence imaging of non-switched and photoswitched mEOS was done by excitation with the 475/575 nm LEDs of the Leica LED8 unit and the emission filters 531/32 and 589/40.

### Organoid Culture, Transduction, Processing, and Imaging

Mouse small intestinal organoids were cultured in Matrigel droplets and Advanced DMEM medium supplemented with HEPES (10 mM), L-glutamine (2 mM), 10% R-Spondin1-conditioned medium, N-2 supplement (1×), B27 supplement (1×), N-acetylcysteine (1 mM), Noggin (100 ng/ml) EGF (50 ng/ml), valproic acid (1 mM), and CHIR-99021 (10 μM) at 37°C and 5% CO_2_. For lentiviral transductions, newly seeded organoids were cultured with stimulation medium for 2 days, containing additionally 50% Wnt3a conditioned medium and 10 mM nicotinamide. Single cells were prepared by treatment with AccuMAX for 10 min at room temperature, resuspended in stimulation medium with additional Y-27632 (10 μM) and mixed with lentiviral supernatants (shTTL, shscr) that were prepared following standard protocols. The cells were then “spinoculated” for 60 min at 600 g in a cell culture plate centrifuge, followed by 6 h of incubation at 37°C and 5% CO_2_. Cells were collected, seeded in Matrigel and cultivated with stimulation medium with additional Y-27632 (10 μM) for 72 h before antibiotic selection with puromycin (0.5 μg/ml) in standard medium was carried out. For imaging and morphological analysis shRNA-expressing organoids were seeded into ibidi 8 well μ Slides and covered with culture medium supplemented with doxycycline (1 μg/ml). Imaging was done for 168 h, every 24 h using a Leica Thunder Imaging Microscope with a 5× objective, recording z-stack tile scans. Z-stacks were transformed into 2D images using the Leica Application Suite X software package’s extended depth of field functionality. Organoid morphological data was obtained by image analysis with OrganoSeg (Borten et al., 2018). Immunostaining was performed after 4% PFA fixation for 1 h at room temperature. The organoids were then permeabilized with 0.2% Triton X-100 for 30 min and blocked in 5% BSA/PBS^++^ for 1 h at room temperature. Primary antibody staining was performed overnight in blocking reagent at 4°C. Secondary antibodies labeled with the indicated Alexa Fluor dyes were added in PBS^++^ for 1 h at room temperature. Cells were washed three times with PBS^++^. Nuclei were stained with Hoechst 33342. Following incubation, cells were washed with PBS^++^ and mounted with Mowiol for fluorescence microscopy. Protein lysates were prepared from shRNA-expressing organoids cultivated in standard culture medium with doxycycline (1 μg/ml) for 96 h. Organoids were disrupted mechanically, Matrigel was washed off with 0.1% BSA in PBS and cells were lysed in RIPA buffer. Lysates were stored at −20°C.

### Organoid RTqPCR

mRNA was reverse transcribed from organoids using the Trifast, reverse transcription protocol and mRNA expression was quantified by RTqPCR using the ΔΔCt method, normalizing to the housekeeping gene Ywhaz. RTqPCR primers: mTtl_fwd1: 5′-CGACGAGAATAGCAGCGTCT-3′, mTtl_rev1: 5′-AGGCTCGTGACCTAGTCTCC-3′, mYwhaz_ fwd: 5′-TTACTTGGCCGAGGTTGCT-3′, mYwhaz_rev: 5′-TGC TGTGACTGGTCCACAAT-3′.

### TER Measurement

To determine transepithelial resistance (TER), equal cell densities of MDCK cells were seeded on six-well plate filter inserts and incubated at 37°C. TER measurement was performed every 24 h using the Millicell ERS-2 Voltohmmeter (Millipore) in triplicates. All TEER values were determined after subtracting the TER of blank inserts. Values were expressed as Ω^⋅^cm^2^.

### Cell Migration Assay

Cell migration was assessed in a wound-healing assay. MDCK cells were cultured until a confluent monolayer was formed (2–3 days). A straight scratch was made using a sterile micropipette tip. Consistent cell-gap widths were measured for each cell line to receive reproducible results. The cells were then washed with PBS three times, and incubated in complete medium at 37°C, 5% CO_2_ and high humidity. Wound closure was monitored over time using a PAULA microscope equipped with the corresponding analysis tools (Personal Automated Lab Assistant, Leica Microsystems).

### Trypsin-Sensitive Detachment Assay

Madin-Darby Canine Kidney cells were seeded on 12-well plates coated or not coated with collagen Type I and cultured for 5 days at 37°C with 5% CO_2_. For de-adhesion, cells were washed with pre-warmed PBS and incubated with 1× trypsin/EDTA (0.05/0.02%) solution for the indicated time points. Detached cells were collected, washed twice with PBS and counted using the Countess Cell Counter (Invitrogen).

### Integrin Uptake Assay

To determine integrin internalization proteins at the cell surface were biotinylated with NHS-SS-biotin for 30 min at 4°C. Then, uptake of proteins was allowed at 37°C for 0 or 30 min before non-internalized proteins were reduced by glutathione. Cells were washed with PBS^++^, collected in lysis buffer by mechanical detachment and incubated at 4°C for 30 min on a rotating platform. After centrifugation (17,000*g* for 15 min), cleared lysates were incubated with neutravidin-agarose beads (NeutrAvidin, Thermo Fisher Scientific) for 2 h at 4°C. Finally, beads were rinsed three times with washing buffer, once with PBS and boiled in SDS/PAGE loading buffer for western blot analysis.

### Proximity Ligation Assay

*In situ* Proximity Ligation Assay (PLA) was performed to analyze the spatial proximity between vinculin and detyrosinated tubulin. Cells were washed twice with PBS^++^, fixed and permeabilized with ice-cold methanol for 5 min. The cells were blocked by adding blocking solution (Duolink) for 1 h at room temperature. Primary antibodies were incubated overnight at 4°C. PLA probes anti-mouse PLUS and anti-rabbit MINUS (Duolink) were added and incubated for 1 h at 37°C. Ligation-reaction and ligase were added, followed by incubation for 30 min at 37°C. Amplification with fluorescent oligonucleotides was carried out for 100 min at 37°C (Duolink, *In Situ* Detection Reagents Orange). Fluorescent emission was investigated by confocal microscopy followed by quantification with the Volocity software package (PerkinElmer).

### Quantifications and Statistical Analysis

Band densities of western blots were measured using LabImage 1D software. Band density values were normalized to GAPDH. The level of detyr-, tyr-, or acetyl-tubulin expression was set to 1. For fluorescence microscopy image analysis intensities of detyr- tubulin-, tyr- tubulin-, TTL-, or SI-positive fluorescence was measured from a minimum of nine images in three experiments using ImageJ. PLA spots were counted from a minimum of 10 pictures in three experiments using Volocity. The Volocity software package was also used to determine number and size of focal adhesions. Cell area, height and fluorescence intensities along predefined lines (line scans) were measured with ImageJ routines.

## Results

### Knockout or Overexpression of TTL Changes Cell Shape and Epithelium Formation

**FIGURE 1:**
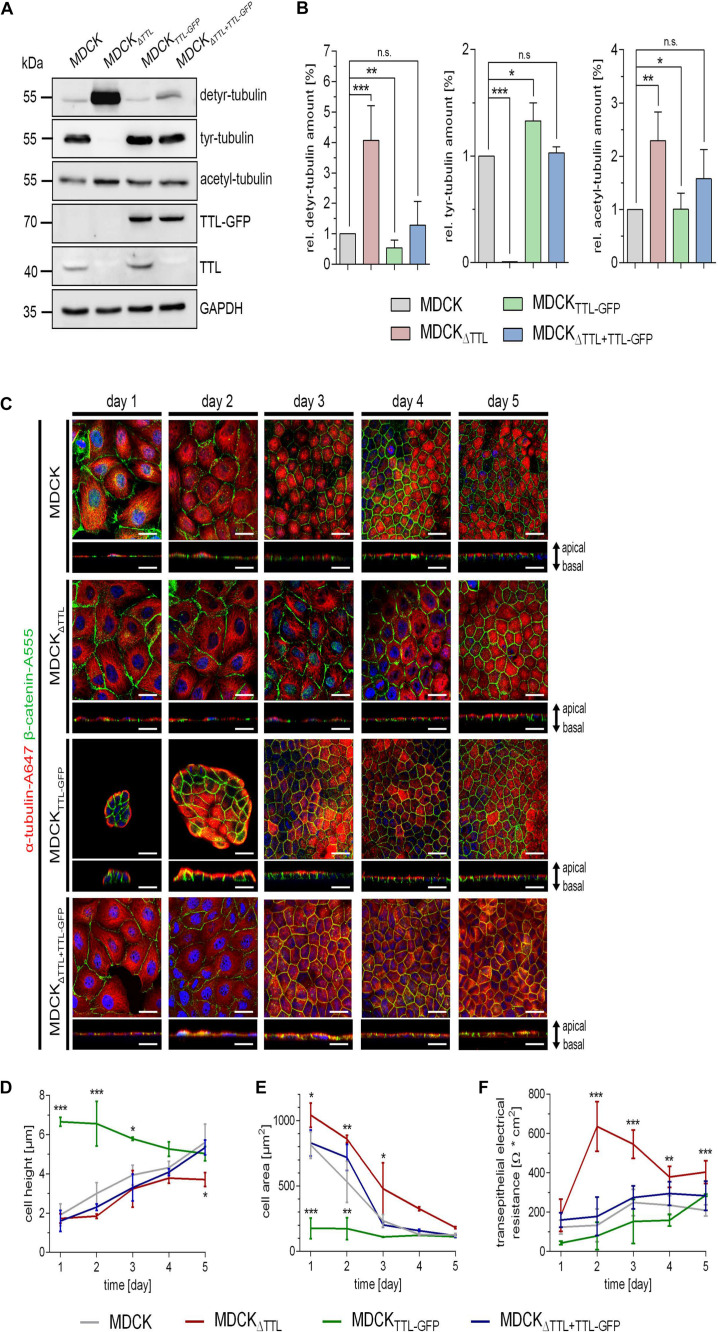
Characterization of MDCK, MDCK_Δ__*TTL*_, MDCK_*TTL–GFP*_, and MDCK_Δ__*TTL* + *TTL–GFP*_ cells. **(A,B)** Cellular levels of detyrosinated, tyrosinated and acetylated tubulin were assessed by western blot analysis of cell lysates from polarized MDCK, MDCK_Δ__*TTL*_, MDCK_*TTL–GFP*_, and MDCK_Δ__*TTL*__+__*TTL–GFP*_ cells. Protein concentrations of the lysates were determined and equal amounts were loaded on each lane of the SDS-PAGE. Relative protein expression was normalized to GAPDH levels. Relative detyrosinated, tyrosinated and acetylated tubulin expression in each cell line as compared to MDCK cells. Quantities from MDCK cells were set as 1. Mean ± SD, *n* = 4. Statistical significance was tested using one-way ANOVA with Dunnet’s comparison (n.s., not significant; *P* < 0.05; **P < 0.01; ***P < 0.001). **(C–E)** At indicated time intervals after filter-seeding MDCK cells were fixed and immunostained with pAb anti-β-catenin (Alexa Fluor 555) and mAb anti-α-tubulin (Alexa Fluor 647). Xy-scans and xz-scans are depicted for each time interval and each cell line. Nuclei are indicated in blue; scale bars, 25 μm. Quantification of cell height **(D)** and cell area **(E)**. Mean ± SD, *n* = 3. Statistical significance was tested with two-way ANOVA and Bonferroni’s post-test (*P < 0.05; **P < 0.01; ***P < 0.001). **(F)** Transepithelial resistance-measurement of MDCK, MDCK_Δ__*TTL*_, MDCK_*TTL–GFP*_, and MDCK_Δ__*TTL*__+__*TTL–GFP*_ cells grown on filters for 6 days. Three filters were used for every cell line and the measurement was performed in triplicates. Mean ± SD, *n* = 3. Statistical significance was tested with two-way ANOVA and Bonferroni’s post-test (***P < 0.001).

At first, expression of TTL was modulated in MDCK cell lines by stable overexpression of a TTL-GFP fusion protein as previously published (MDCK_*TTL–GFP*_) (Zink et al., 2012) or by gene-knockout (MDCK_Δ__*TTL*_). Under constantly enhanced TTL-GFP levels the amount of detyr-tubulin was decreased in MDCK_*TTL–GFP*_ cells (Figures 1A,B). Opposing effects were revealed in MDCK_Δ__*TTL*_ cells. These cells were depleted in tyr-tubulin and enhanced in detyr-tubulin levels. In parallel, acetylated microtubule quantities were also enhanced in MDCK_Δ__*TTL*_ cells and declined in these cells if TTL-GFP was expressed. This suggests that a complete removal of TTL intracellularly accumulates detyrosinated and acetylated microtubules. Expression of TTL-GFP in MDCK_Δ__*TTL*_ cells consistently reduced acetylated and detyr-tubulin and restored tyr-tubulin quantities to almost standard levels in MDCK cells. Alterations in microtubule-disposition and the morphogenesis of MDCK, MDCK_Δ__*TTL*_, MDCK_*TTL–GFP*_, and MDCK_Δ *TTL+TTL–GFP*_ cells were assessed by immunofluorescence analysis following up to 5 days of epithelial differentiation. One day after plating, line scan analysis of α-tubulin intensities revealed that a central, perinuclear packaging of microtubules, which was very prominent in MDCK_Δ__*TTL*_ cells, was shifted to a peripheral accumulation in MDCK_*TTL–GFP*_ cells (Figure 1C and Supplementary Figure 1A). Furthermore, early after seeding MDCK_*TTL–GFP*_ cells showed a much higher tendency to join into islands of 6–12 columnar cells than MDCK or MDCK_Δ__*TTL*_ cells (Figure 1C). MDCK_Δ__*TTL*_ cells had an outspread and flat morphology and slowly reached a height of only about 4 μm after 5 days in culture, which was rescued by TTL-GFP-expression (Figure 1D). The apparent differences in cell morphology have also been quantified by cross section measurements of the four cell lines showing that MDCK_Δ__*TTL*_ cells had the largest and MDCK_*TTL–GFP*_ cells the smallest diameter early after seeding (Figure 1E). Nevertheless, all cell lines ended up with similar cell areas following epithelial differentiation. Interestingly, the proliferation rate of MDCK_Δ__*TTL*_ cells was significantly slower than that of MDCK cells expressing the TTL enzyme (Supplementary Figures 1B,C). Major alterations between the cell lines during morphogenesis were further detected by measurement of the TER, which provides an indication of epithelial barrier integrity. Figure 1F shows for MDCK_Δ__*TTL*_ cells an instant TER-increase to a maximum after 2 days following seeding onto filter inlets indicating that they early assembled into flat tight monolayers. On the other hand, the TER of MDCK, MDCK_*TTL–GFP*_, and MDCK_Δ *TTL+TTL–GFP*_ cells gradually ascended to reach a maximum after 4–6 days. Thus, the flat morphology of MDCK_Δ__*TTL*_ cells seemed to facilitate instantaneous monolayer formation even if the proliferation rate was low. However, they had not reached full height and were therefore not completely differentiated at that stage. In contrast, MDCK_*TTL–GFP*_ cells had a prematurely differentiated morphology (Zink et al., 2012), which suggests that the differentiated epithelial architecture was stabilized if cellular quantities of detyr-tubulin were reduced.

To estimate if tubulin-tyrosination in general modulates the differentiation of epithelial cells we extended our analysis to cells from a different organ. Therefore, TTL-GFP was stably overexpressed in epithelial colorectal Caco-2 cells. Posttranslational tubulin modifications in Caco-2 cells following TTL-GFP-overexpression were similar to the pattern observed in MDCK cells with a significant decrease in detyr-tubulin (Supplementary Figures 1D,E). In analogy to MDCK cells TTL-overexpressing Caco-2_*TTL–GFP*_ cells were taller and had a smaller diameter than Caco-2 cells early after seeding (Supplementary Figures 1F,G), thus indicating that the detyr-/tyr-tubulin equilibrium determined the architecture of intestinal as well as kidney epithelial cells.

**FIGURE 2:**
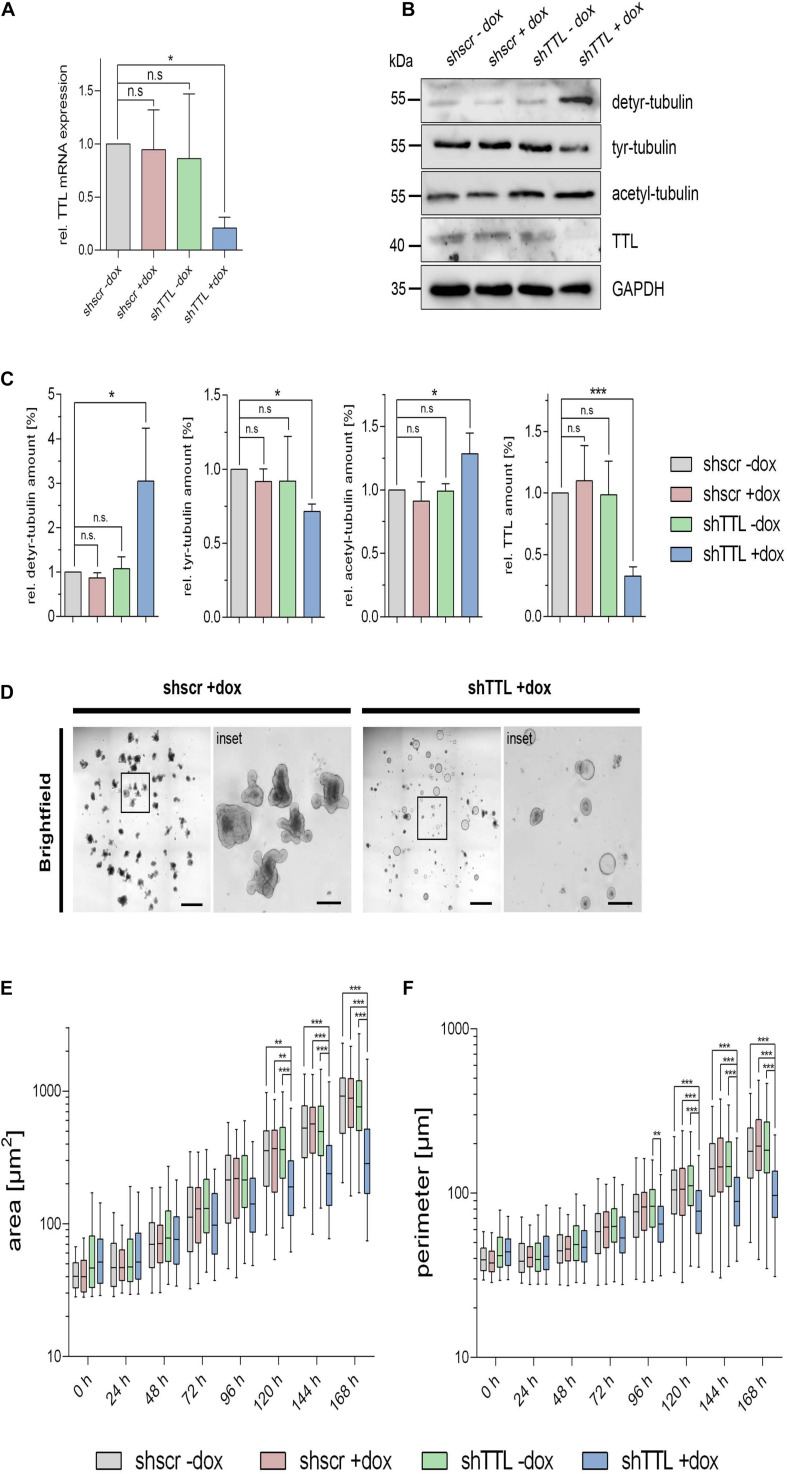
Altered morphogenesis of small intestinal organoids following TTL-knockdown. **(A)** TTL mRNA expression levels were measured by RTqPCR in control organoids expressing a scrambled (shscr) shRNA and TTL-knockdown organoids expressing shRNA targeting TTL (shTTL) after 96 h of culture in the presence (+dox) or absence (–dox) of doxycycline. Values were calculated relative to scr –dox control and plotted as mean ± SD, *n* = 3. Statistical significance was tested using one-way ANOVA with Dunnet’s comparison. **(B,C)** Cellular levels of detyr-tubulin, tyr-tubulin, acetyl-tubulin, and TTL were assessed by immunoblot analysis of cell lysates from knockdown and control organoids. Relative protein expression was quantified and normalized to GAPDH levels. Quantities from scr –dox control organoids were set as 1. Mean ± SD, *n* = 3. Statistical significance was tested using one-way ANOVA with Dunnet’s comparison (n.s., not significant; *P < 0.05; ***P < 0.001). **(D–F)** Representative images of TTL-depleted organoids after 168 h of culture in the presence of doxycycline. Images were taken with a 5× objective. Scale bars overview: 100 μm, scale bars inset: 25 μm. Quantification of TTL and scr control organoid size and perimeter **(E,F)**. Mean ± SD, *n* = 3, 15–20 organoids per experiment. Statistical significance was tested with two-way ANOVA and Bonferroni’s post-test (**P < 0.01; ***P < 0.001).

**FIGURE 3:**
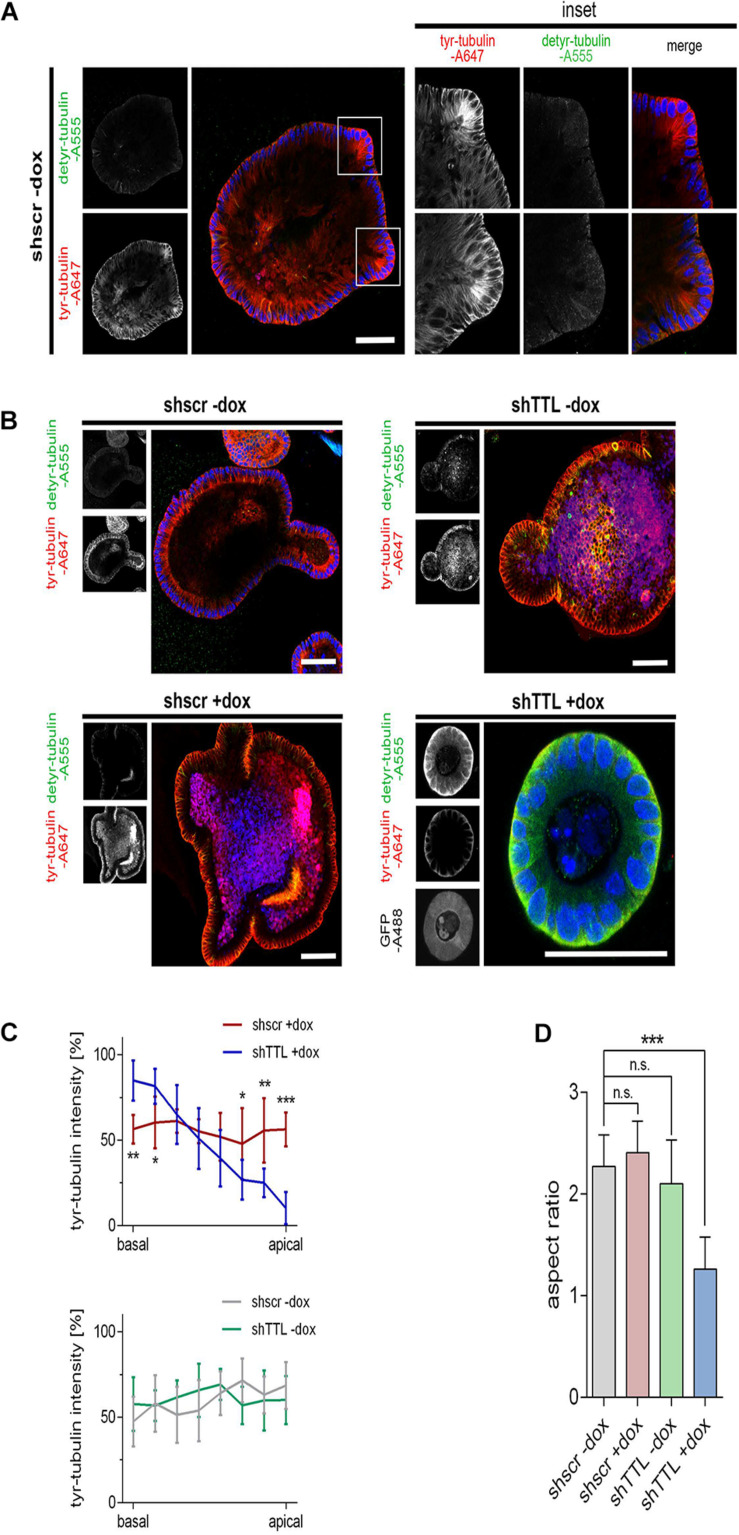
Cell flattening in TTL-KD small intestinal organoids. **(A,B)** Control organoids expressing sh scrambled (shscr ±dox, shTTL –dox) and TTL-knockdown organoids expressing shRNA targeting TTL (shTTL +dox) were fixed after 96 h of culture in the presence (+dox) or absence (–dox) of doxycycline. GFP staining positively correlates with shTTL expression. The samples were stained with antibodies against detyr- (Alexa Fluor 555) and tyr-tubulin (Alexa Fluor 647). Inset boxes in **(A)** show budding areas with elevated quantities of tyr-tubulin. Scale bars: 60 μm. **(C)** Tyr-tubulin intensities were measured by line scan analysis from the basal to the apical cell pole. Ten cells were analyzed from each experiment. Mean ± SD, *n* = 3. Statistical significance was tested with two-way ANOVA and Bonferroni’s post-test (*P < 0.05; **P < 0.01; ***P < 0.001). **(D)** Aspect ratios of control organoids and TTL-knockdown organoids. Mean ± SD, *n* = 3. Statistical significance was tested with Student’s *t*-test (n.s., not significant; ***P < 0.001).

We now switched from a 2D cell culture model to 3D culture and monitored the organogenesis of small intestinal organoids that were TTL-depleted by inducible gene-knockdown. Therefore, crypts were isolated from mouse intestine and infected with lentiviral vectors for non-specific scrambled or specific TTL-shRNA production. TTL-knockdown efficiency after doxycycline-induction was verified by RT-PCR and immunoblot analysis (Figures 2A–C). Immunoblot analysis also revealed that detyr-tubulin as well as acetylated tubulin were significantly increased in TTL-depleted organoids. Following 7 days of incubation, Matrigel-embedded organoids in the control group formed typical finger-like structures budding outward, while TTL-depleted crypts made non-branched spheroids of obviously reduced size (Figures 2D,E). The decrease in organoid-branching following TTL-knockdown was further reflected by perimeter-reduction as quantified in Figure 2F. Especially the budding structures of control organoids showed intense tyr-tubulin signals by whole mount immunofluorescence staining (Figure 3A). The tyr-tubulin distribution from apical to basal shifted toward the basal cell pole if TTL was depleted (Figures 3B,C). On the other hand, a detyr-tubulin rise following TTL-knockdown goes along with a reduction in cell height and resulted in a cuboidal cell shape with an average height:width aspect ratio of 1.32 in contrast to the average aspect ratio of 2.41 from columnar cells in control organoids (Figures 3B,D). These morphological changes are reminiscent of consequences following knockout or overexpression of TTL in Caco-2 and MDCK cells. Thus, a decrease in TTL-expression seems to redistribute the polar distribution of tyr-tubulin, to flatten epithelial cells in 2D and 3D with a broadened basal membrane and to affect organoid formation as evidenced by the lack of crypt-like budding structures formed in growing TTL-knockdown mini-guts.

**FIGURE 4:**
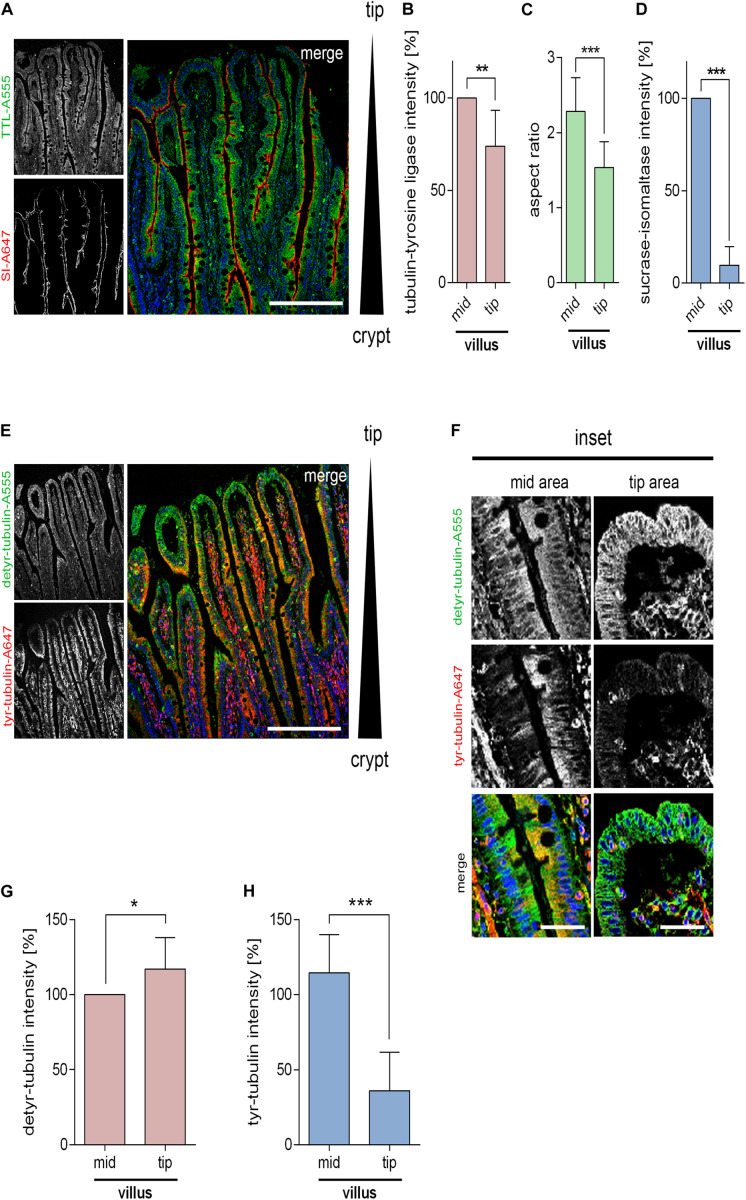
Distribution of detyr-tubulin, tyr-tubulin, and TTL along intestinal villi. **(A)** Cross-sections of human small intestinal villi stained with antibodies against TTL (Alexa Fluor 555) and sucrase isomaltase (SI; Alexa Fluor 647). Images were taken with a 20× objective. Nuclear counterstaining with Hoechst 33342 is indicated in blue. Scale bars: 300 μm. Quantification of TTL staining **(B)**, aspect ratios **(C)**, and SI-intensity **(D)** of villus tip and mid-villus areas. Mean ± SD, *n* = 3 (nine pictures). Statistical significance was tested with Student’s *t*-test (*P < 0.05, **P < 0.01). **(E)** Cross-sections of human small intestinal villi stained with antibodies against detyr- (Alexa Fluor 555) and tyr-tubulin (Alexa Fluor 647). Images were taken as indicated above. Scale bar: 300 μm. **(F)** Magnified mid-villus areas or villus tips from detyr- and tyr-tubulin-stained cross sections. Scale bar: 50 μm. **(G,H)** Quantification of detyr- and tyr-tubulin staining of villus tip and mid-villus regions. Mean ± SD, *n* = 3 (10 pictures). Statistical significance was tested with Student’s *t*-test (n.s., not significant; *P < 0.05, **P < 0.01; ***P < 0.001).

We then analyzed the TTL-distribution along the crypt-villus axis in paraffin embedded human small intestinal samples by immunofluorescence. Figures 4A,B shows a continuous expression of TTL along intestinal villi and an abrupt decline in the villus tip areas. Concurrently, enterocytes along the villus have a columnar shape with an average aspect ratio of 2.4 in contrast to enterocytes at the villus tip with an aspect ratio of 1.54 (Figure 4C). This aspect ratio pattern is reminiscent of crypts and villi in developing mouse intestine (Sumigray et al., 2018). The detyr- and tyr-tubulin distribution was also determined by immunofluorescence in human small intestine using corresponding antibodies. Along the crypt-villus axis detyr-tubulin quantities rose from the villus area up to the villus tip. On the contrary, the amount of tyr-tubulin and the brush border enzyme SI, a hydrolase expressed in differentiated enterocytes, declined along this axis (Figures 4D–H). A decline of tyr-tubulin and SI was most dramatic at the extreme tip in the so-called extrusion zone (Williams et al., 2015). Here, cells showed only faint TTL-staining. In conclusion, data from epithelial cell lines, small intestinal organoids and the small intestine altogether indicate that epithelial morphogenesis is determined by TTL-expression and that a loss of TTL is accompanied by cell flattening and a broader basal cell membrane.

### MDCK_Δ__*TTL*_ Cells Adhere Strongly and Migrate Fast

**FIGURE 5:**
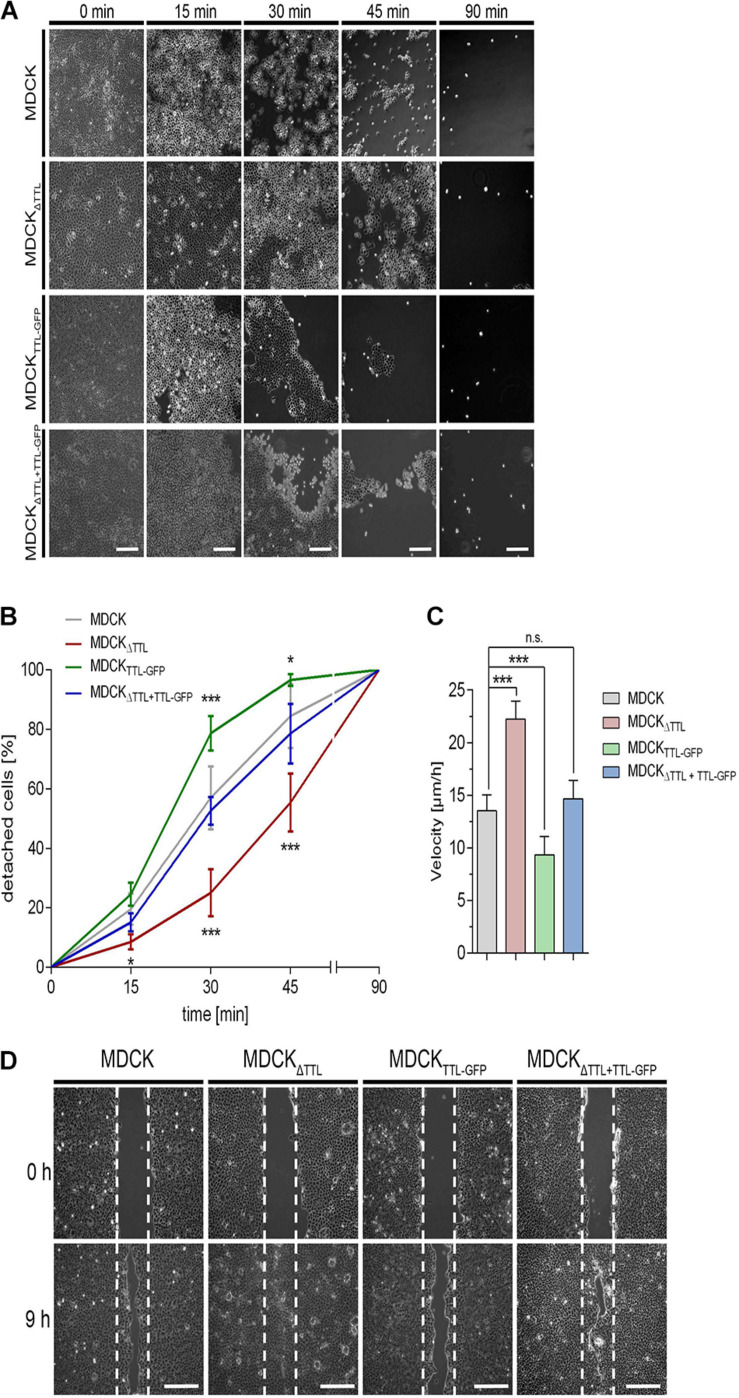
Influence of tubulin detyrosination on cell adhesion and migration. **(A,B)** A trypsin-sensitive cell detachment assay was performed to measure the strength of cell attachment. Confluent cell layers of MDCK, MDCK_Δ__*TTL*_, MDCK_*TTL–GFP*_, and MDCK_Δ__*TTL*__+__*TTL–GFP*_ cells were washed with PBS and then incubated with warm trypsin/EDTA (0.05/0.02%) for indicated time intervals. Scale bars: 100 μm. Quantitative results of the detachment assay are depicted as percentage of detached cells **(B)**. Mean ± SD, *n* = 4. Statistical significance was tested with two-way ANOVA and Bonferroni’s post-test (n.s., not significant; *P < 0.05; **P < 0.01; ***P < 0.001). **(C,D)** Confluent monolayers of MDCK cells were scratch wounded to analyze migration. Cells were recorded at 0, 3, 6 (3 and 6 not shown) and 9 h post-scratching. **(C)** Mean cell migration velocity was calculated. Mean ± SD, *n* = 5. Statistical significance was tested with Student’s *t*-test (n.s., not significant; ***P < 0.0001). **(D)** Images recorded immediately after (0 h) or 9 h post scratching are depicted. White dotted lines indicate the wound borders at the beginning of the assay. Scale bars: 100 μm.

Based on the observed cell flattening and expansion of the basal part of the cells we assumed that cell adhesion to the extracellular matrix was affected. Hence, we examined the adhesion efficiency of our MDCK cell lines. Trypsin-induced de-adhesion dynamics of MDCK, MDCK_Δ__*TTL*_, MDCK_*TTL–GFP*_, and MDCK_Δ *TTL+TTL–GFP*_ cells was determined on non-coated (Figures 5A,B) or collagen-coated petri dishes (Supplementary Figures 2A,B). MDCK_*TTL–GFP*_ cells rapidly detached from the underlying surface in the presence of trypsin. On the contrary, MDCK_Δ__*TTL*_ cells strongly adhered to remain about 50% confluent following 45 min of trypsin treatment. Overexpression of TTL-GFP normalized the adhesion efficiency in MDCK_Δ *TTL+TTL–GFP*_ cells. Opposing effects following TTL-knockdown or -overexpression were further reflected in the migration characteristics of the two cell lines. Here, we performed scratch wound healing assays, where a wound gap is created by scratching in the cell monolayer. Healing of this gap by cell migration and growth toward the center of the gap was then monitored and quantified (Figures 5C,D). MDCK_Δ__*TTL*_ cells migrated significantly faster than MDCK, MDCK_*TTL–GFP*_, and MDCK_Δ *TTL+TTL–GFP*_ cells, with MDCK_*TTL–GFP*_ cells showing the slowest migration pattern. In accordance with MDCK cells, TTL-overexpressing Caco-2_*TTL–GFP*_ cells adhered less and migrated slower than Caco-2 cells (Supplementary Figures 2C–E).

**FIGURE 6:**
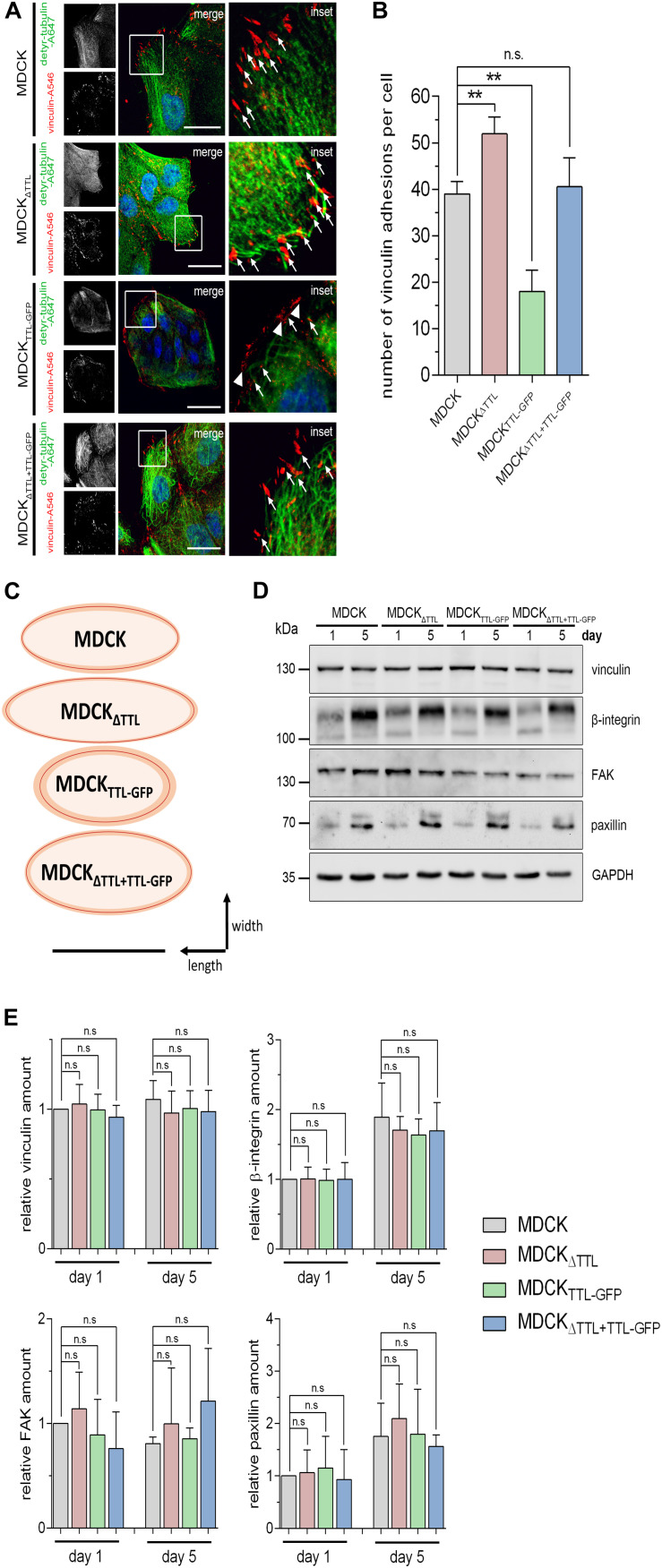
Correlation between tubulin detyrosination and the number/size of vinculin-positive focal adhesions. **(A–C)** Subconfluent MDCK, MDCK_Δ__*TTL*_, MDCK_*TTL–GFP*_, and MDCK_Δ__*TTL*__+__*TTL–GFP*_ cells were immunostained with pAb anti-detyr-tubulin (Alexa Fluor 647) and mAb anti-vinculin (Alexa Fluor 546). Arrows indicate colocalization of detyrosinated microtubule and vinculin. These focal adhesions are orientated in the direction of migration. Arrowheads indicate vinculin-positive focal adhesions arranged at the edge of the isolated islands typically formed by MDCK_*TTL–GFP*_ cells. Scale bar: 25 μm. **(B)** Quantification of vinculin-positive focal adhesions per cell. Mean ± SD, 8–10 cells per experiment, *n* = 3 independent experiments. Statistical significance was tested using one-way ANOVA with Dunnet’s comparison (n.s., not significant; **P < 0.01). **(C)** Schematic diagram showing the average size and shape from vinculin-positive focal adhesions in MDCK, MDCK_Δ__*TTL*_, MDCK_*TTL–GFP*_, and MDCK_Δ__*TTL*__+__*TTL–GFP*_ cells. Shape of focal adhesions is shown as ovals with best fit around length and width. Average sizes are indicated by red lines, SD is depicted in orange. A total of 15–20 focal adhesions were measured per experiment. Scale bar: 1 μm. **(D,E)** Lysates of subconfluent and confluent MDCK, MDCK_Δ__*TTL*_, MDCK_*TTL–GFP*_, and MDCK_Δ__*TTL*__+__*TTL–GFP*_ cells were analyzed by immunoblot with antibodies directed against vinculin, β1-integrin (CD29), focal adhesion kinase (FAK), and paxillin. Equal amounts (20 μg) of lysates were loaded. GAPDH served as a loading control. **(E)** Relative quantities were normalized to GAPDH levels in cell lysates. Mean ± SD, *n* = 3 for subconfluent, and *n* = 4 for confluent cell lysates independent experiments. Statistical significance was tested using one-way ANOVA with Dunnet’s comparison (n.s., not significant).

Cell migration essentially depends on the assembly and disassembly of focal adhesions, which build up physical connections between the extracellular matrix and the actin cytoskeleton through transmembrane receptor integrins (Ridley et al., 2003). Number and size of focal adhesions were quantified in subconfluent MDCK, MDCK_Δ__*TTL*_, MDCK_*TTL–GFP*_, and MDCK_Δ *TTL+TTL–GFP*_ cells using the vinculin marker (Figures 6A–C). In agreement with observations from fibroblasts (Gundersen and Bulinski, 1988; Palazzo et al., 2004) the detyr-tubulin-enriched tubules were oriented toward the leading edge and their ends were in close proximity to vinculin-positive focal adhesions. We counted significantly more focal adhesions in MDCK_Δ__*TTL*_ than in MDCK or MDCK_Δ *TTL+TTL–GFP*_ cells (Figure 6B). The lowest number of focal adhesions was found in MDCK_*TTL–GFP*_ cells. These observations were confirmed if paxillin was immunostained as focal adhesion marker protein (Supplementary Figures 3A–C). In addition, focal adhesions were the smallest in MDCK_*TTL–GFP*_ cells and the largest in MDCK_Δ__*TTL*_ cells, which migrate faster (Figure 6C). This nicely corresponds to the idea that the mean size of focal adhesions predicts migration velocity (Kim and Wirtz, 2013).

In search for the reason for these size differences of focal adhesions, we first analyzed the expression patterns of the focal adhesion components vinculin, β1-integrin (CD29), focal adhesion kinase (FAK) and paxillin in subconfluent or confluent MDCK, MDCK_Δ__*TTL*_, and MDCK_*TTL–GFP*_ cells by immunoblot (Figures 6D,E). However, quantification of the corresponding bands did not reveal gross changes in their expression pattern following modulation of TTL. This suggests that an increase in focal adhesion size and quantity is not based on a general increase in these focal adhesion components in MDCK_Δ__*TTL*_ cells. It rather seems that following TTL-knockout the organization and assembly of focal adhesions is altered.

### Dynamics of Focal Adhesion Compounds in MDCK_Δ__*TTL*_ Cells

**FIGURE 7:**
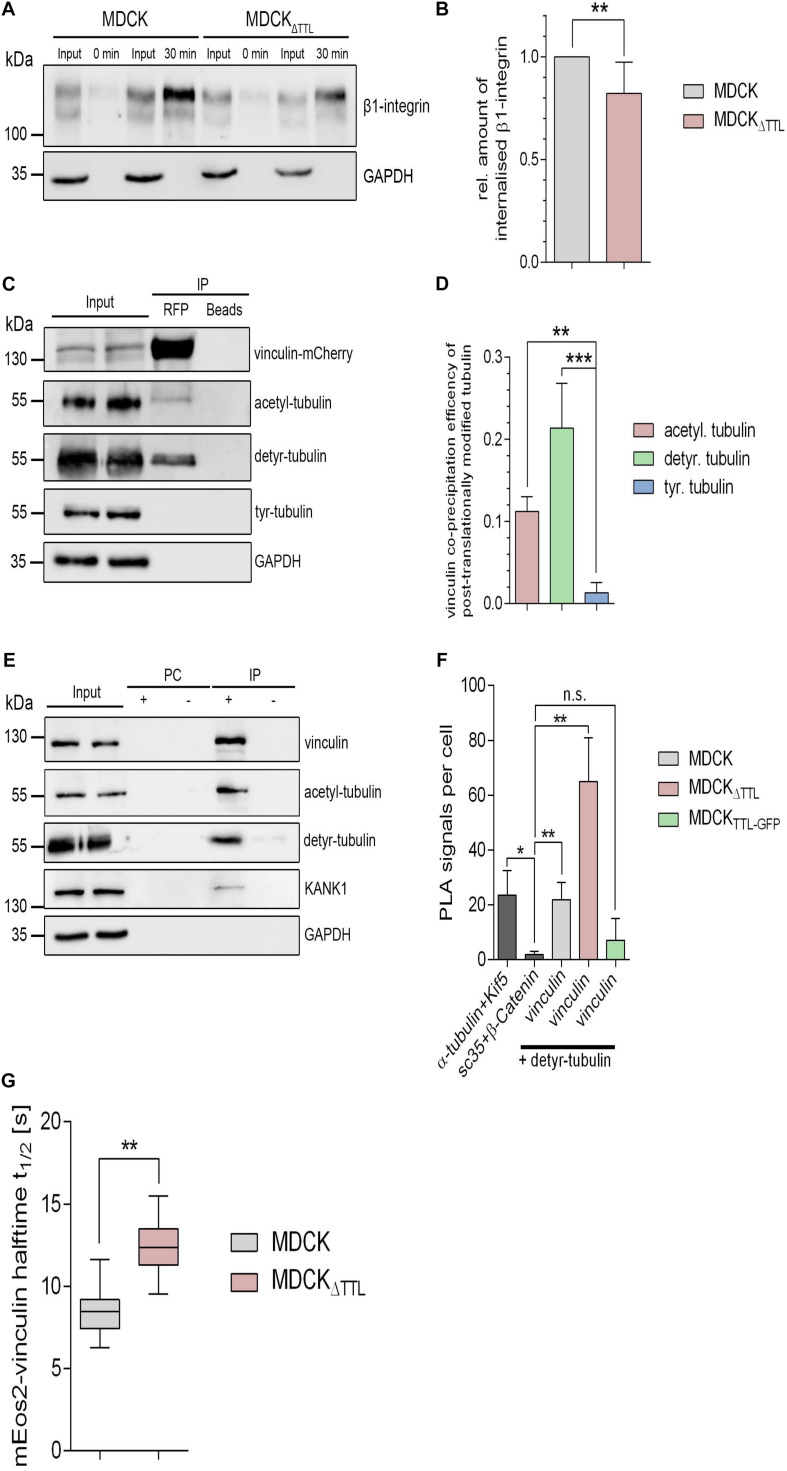
Integrin-internalization and vinculin-interaction followingmodulation of TTL. **(A,B)** Membrane proteins of MDCK and MDCK 1 TTL cells were biotinylated with NHS-SS-biotin. Endocytosis of biotin-labeled membrane proteins was allowed for 0 or 30 min. Biotin label of non-internalized proteins was removed by reduced glutathione. Cells were lysed, biotinylated proteins were precipitated with neutravidin-beads, and precipitates were analyzed by immunoblot against β1-integrin and GAPDH. Corresponding lysate fractions are indicated as input. GAPDH was used as internal control. **(B)** The amount of internalized β1-integrin was normalized by the total integrin quantities in the input. Mean ± SD, *n* = 3. Statistical significance was tested with Student’s *t*-test (**P < 0.01). **(C)** MDCK cells transfected with vinculin-mCherry were lysed 36 h post-transfection. Cell lysates were incubated with RFP-Trap beads or blocked agarose beads (negative control). Western blots were incubated with anti- vinculin-, anti- acetyl-, anti- detyr-, anti-tyr-tubulin, or anti GAPDH antibodies. **(D)** Quantification of the co-precipitation efficiencies of posttranslationally modified α-tubulin from three independent experiments. The efficiency was normalized by the total quantities of each polypeptide in the input. Statistical significance was tested with Student’s unpaired *t*-test (*P < 0.05). **(E)** MDCK_Δ__*TTL*_ cell lysates were incubated with anti-vinculin antibodies followed by precipitation with agarose beads. Precipitates were analyzed by immunoblot using antibodies directed against acetyl, detyr-tubulin, KANK1, GAPDH, and vinculin. Representative results, *n* = 3 independent experiments. PC, pre-clearing; IP+, immunoprecipitation using vinculin antibodies and agarose beads; IP–, immunoprecipitation using agarose beads without antibodies. **(F)** Proximity ligation assays (PLA) were performed to analyze proximal association of detyr-tubulin and vinculin in MDCK, MDCK_Δ__*TTL*_, and MDCK_*TTL–GFP*_ cells. Quantification of proximity ligation events as shown in Supplementary Figure 4. PLA signals were significantly increased compared to the negative control of the nuclear speckles-marker Sc35 and β-catenin. Proximity between α-tubulin and the microtubule motor protein Kif5 was used as positive control. Statistical significance was tested using one-way ANOVA with Tukey’s multi comparison (n.s., not significant; *P < 0.05; **P < 0.01), *n* = 3 independent experiments. **(G)** Halftime of photoactivated vinculin mEOS at focal adhesions as recorded in Supplementary Figure 5. *n* = 3. Statistical significance was tested with two-way ANOVA and Bonferroni’s post-test (**P < 0.01).

We focused on two aspects in focal adhesion turnover to address this point. At first, we checked a putative role of posttranslationally modified microtubules in the endocytic uptake of integrins. Therefore, β1-integrin was labeled with a reducible biotin conjugate in MDCK and MDCK_Δ__*TTL*_ cells. Internalization of biotin-labeled membrane proteins was allowed for 30 min at 37°C. Subsequently, biotin was removed from non-internalized biotinylated proteins by glutathione. Precipitation of internalized biotinylated proteins with neutravidin-beads and immunoblot analysis revealed a significant decrease in β1-integrin internalization in MDCK_Δ__*TTL*_ cells(Figures 7A,B and Supplementary Figures 3D,E). This suggests that recycling of integrin adhesion receptors is diminished if the detyr-tubulin-concentration is elevated, which would prolong their residence time at the plasma membrane.

Secondly, recent evidence indicates that microtubules are coupled to focal adhesions via links formed by scaffolding polypeptides (Rafiq et al., 2019). It thus seems plausible that detyrosinated microtubules directly or indirectly interact with vital focal adhesion components. We therefore determined pulldown of acetylated, detyr-, or tyr-tubulin by vinculin-mCherry from MDCK cell lysates. Vinculin-mCherry as well as endogenously expressed vinculin was co-precipitated together with detyr- and acetylated tubulin (Figures 7C–E). Moreover, the focal adhesion adapter kidney ankyrin repeat-containing protein 1 (KANK1) and β1-integrin were pulled down by vinculin (Figure 7E and Supplementary Figures 3F–I). Interestingly, pulldown of these two focal adhesion components was significantly increased in MDCK_Δ__*TTL*_ cells, which can be explained by an increased number and size of focal adhesion plaques under elevated detyr-tubulin concentrations. Biochemical interaction between detyr-tubulin and focal adhesion components was confirmed by spatial proximity between detyr-tubulin and vinculin in proximity ligation assays (PLA). Here, we found that vinculin and detyr-tubulin frequently accrued to a maximum distance of 40 nm within the cytosol (Figure 7F and Supplementary Figure 4). This suggests that detyrosinated microtubules are closely connected to the large protein complex of focal adhesions by direct or indirect interaction. The next step was to find out if enhanced detyr-tubulin levels in MDCK_Δ__*TTL*_ cells affect the residence time of vital polypeptides at focal adhesions. Here, we traced a photoactivatable vinculin-mEOS2 fusion protein, which was photoconverted at individual focal adhesions using 405 nm laser excitation. When transfected into MDCK_Δ__*TTL*_ cells the signal intensity of photoconverted vinculin-mEOS2 declined significantly slower than in MDCK cells (Figure 7G and Supplementary Figure 5), thus indicating that high concentrations of detyr-tubulin in MDCK_Δ__*TTL*_ cells positively affect vinculin-residence at focal adhesions.

Considered together, the prolonged residence time of β1-integrin at the plasma membrane and a longer persistence of vinculin at cell adhesion foci following TTL-knockout strongly argues in favor of a central role of TTL in focal adhesion disintegration and in epithelial cell morphology.

## Discussion

In this study we show, that in the absence of TTL epithelial cells alter their morphology as characterized by a loss of cell elongation and stretching of the cell basis, which is facilitated by elevated persistence of focal adhesions for cell attachment to the extracellular matrix.

Focal adhesions often appear associated with microtubules. In migrating cells detyrosinated or so called “pioneer microtubules” with a characteristic decrease in catastrophe frequency are oriented toward the leading edge (Wittmann et al., 2003). This orientation is mediated by the formin mDia in NIH 3T3 cells (Palazzo et al., 2001). Others found evidence for direct spatial interaction between microtubules and adhesion sites (Kaverina et al., 1998). These microtubules were stabilized at vinculin-contact sites and destabilized in cells lacking these focal contacts. Moreover, Palazzo et al. (2004) reported that cell adhesion is required to form and maintain stable microtubules. Their observations suggest that microtubules are stabilized at the leading edge of migrating cells by an integrin-FAK-mediated signaling cascade. Microtubules may also facilitate the targeting of clathrin to focal adhesions for integrin-uptake and focal adhesion disassembly in migrating cells (Ezratty et al., 2009). In addition, they serve as tracks for secretory vesicles and protein secretion in the vicinity of focal adhesions (Fourriere et al., 2019). Recently, Bance et al. (2019) published that microtubule acetylation promotes Rab6-positive vesicle fusion at focal adhesions. They found that depletion of the major tubulin acetyltransferase αTAT1 in primary astrocytes strongly decreased microtubule acetylation and cell migration speed. This positive correlation between the acetylation rate of microtubules and cell migration is analogous to our observations of detyr-tubulin enriched microtubules in epithelial cells. Acetylated tubulin concentrations are additionally elevated following TTL-knockdown in organoids or -knockout in MDCK cells, which argues for the promotion of cell migration by acetylated microtubules. The question if acetylated or detyrosinated microtubules or even a combination of both are leading to the observed effects in cell migration and morphology can thus not definitively be assigned. It is interesting that transient TTL-GFP-overexpression to reduce detyr-tubulin did not significantly affect astrocyte migration (Bance et al., 2019). This is different in epithelial Caco-2 or MDCK cells stably expressing TTL-GFP, which migrate slightly but significantly slower. Here, we found that especially the loss of TTL in MDCK_Δ__*TTL*_ cells dramatically increases cell adhesion, number and size of focal adhesions and migration speed. These modifications are accompanied by alterations in cell morphology and monolayer formation, which likely reflects cellular immaturity. On the other hand, enhanced TTL levels prematurely elongate epithelial cells into a columnar shape and thus stabilize the polarized epithelial architecture (Quinones et al., 2011; Zink et al., 2012). Consequently, after an initial boost the number of detyrosinated microtubules drops down in polarized epithelial cells that have passed through the differentiation process. In agreement with this TTL-KO fibroblasts with increased detyrosinated tubules lose their polarization (Peris et al., 2006) and TTL-KO neurons show abnormal axonal differentiation (Erck et al., 2005). These observations are in line with the *in vivo* expression pattern of TTL along the small intestinal villus, which indicates that enterocytes wearing off their polarized architecture at the villus tip decrease TTL-expression and tyr-tubulin quantities. A diminished enterocyte height has been described for the particular pathologic conditions in coeliac disease patients (Tabbaa et al., 1994) and in patients receiving cancer chemotherapy (Keefe et al., 2000), both of which impair intestinal absorption. Cell height is also decreased in the last nephron sections of mouse or rat kidneys as assessed by ultrastructural analysis of cells lining proximal tubules, which is relevant to the renal transport physiology (Dorup and Maunsbach, 1997; Zhai et al., 2006). This suggests that the morphological changes induced in intestinal or renal epithelial cells by removal or overexpression of TTL affect the functionality of the whole organ.

Variations in the expression level of TTL have been previously described. Low levels of this enzyme and an increase in detyr-tubulin is a common feature of several types of cancer cells (Mialhe et al., 2001; Kato et al., 2004; Soucek et al., 2006; Rong et al., 2017). The expression pattern in tumor tissues is thereby linked to tumor aggressiveness with a favorable prognosis if TTL expression is high. Conversely, down-regulated TTL expression in fibroblasts promotes tubulin detyrosination and tumor growth in mice (Rong et al., 2017). Low levels of TTL are furthermore critical for a process of cell-cell fusion called trophoblast syncytialization since knockdown of TTL restores the fusion capacity of cytotrophoblast cells derived from preeclamptic placentae (Wang et al., 2019). In addition, tubulin detyrosination is promoted in cells overexpressing VASH2, the enzyme that removes the C-terminal tyrosine of α-tubulin (Iida-Norita et al., 2019). VASH2 overexpression results in strongly increased migration of human pancreatic cancer cells. This indicates that either downregulation of TTL and/or upregulation of VASH2 enhance the cellular detyr-tubulin concentration, which is critical for cell adhesion.

The question is how the formation of detyr-tubulin-enriched tubules is related to highly spatially controlled focal adhesion dynamics. Microtubule disruption in general modulates the size of paxillin- or vinculin-containing focal adhesions (Bershadsky et al., 1996; Zhang et al., 2010). The failure to form discrete focal adhesions goes along with a marked decrease of detyr-tubulin and ambiguous anterior–posterior polarity in migrating fibroblasts (Morioka et al., 2009). A polarity-loss can be explained by abnormal high microtubule turnover in these cells, which would affect directional persistence in migration. Another aspect would be that detyr-tubulin-enriched tubules facilitate the assembly kinetics of focal adhesions or prevent their disassembly. This scenario is favored by diminished recycling of integrin adhesion receptors or a positive correlation between focal adhesion augmentation and the quantity of detyr-tubulin-enriched tubules in our experiments. Recent work indicates that KANK proteins are required for targeting microtubules to focal adhesions (Bouchet et al., 2016; Rafiq et al., 2019). KANK-mediated microtubule coupling suppresses the ability of the Rho nucleotide exchange factor GEF-H1 to be activated by release from microtubules, a condition that limits the growth of focal adhesions. Increased quantities of detyrosinated microtubules would uncouple this regulatory interplay since GEF-H1 does not seem to bind to detyrosinated microtubules (Nagae et al., 2013). It is thus tempting to speculate that in MDCK_Δ__*TTL*_ cells the loss of tyrosinated microtubules at focal adhesions releases and thereby activates GEF-H1 to initiate a RhoA/Rho kinase/myosin light chain signaling pathway that finally promotes focal adhesion augmentation.

The nanoscale architecture and the relative disposition of a multiplicity of focal adhesion components likewise defines the dynamics of these complex structures (Horton et al., 2015). Our study suggests preferential interaction of detyrosinated above tyrosinated microtubules with an intricate network of KANK1, integrin, vinculin, and most likely additional focal adhesions components. As assessed by super resolution microscopy, KANK1 is localized predominantly at the focal adhesion rim (Stubb et al., 2019), where it interacts with the mechanosensitive integrin-associated adaptor talin. This controls focal adhesion dynamics and also serves as a binding hub for vinculin. Accordingly, knockdown of KANK1 expression using siRNA led to smaller cornerstone focal adhesions at the edge of cell colonies (Stubb et al., 2019). The question if this reduction in focal adhesion size is linked to a reduced microtubule coupling remains to be elucidated.

## Conclusion

**FIGURE 8:**
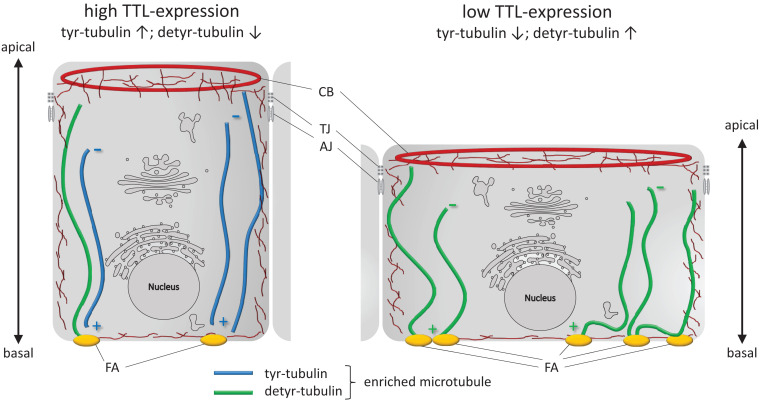
Schematic illustration of cell adhesion and flattening following TTL depletion in epithelial cells during monolayer formation. Cultured columnar epithelia have vertical microtubules of uniform polarity with the minus-ends facing the apical surface. Interaction of microtubules enriched in detyr-tubulin and focal adhesions (FA) extend the basal membrane surface area. CB, contractile belt; TJ, tight junction; AJ, adherens junction; ↑, upregulation; ↓, downregulation.

To conclude, we summarize changes in cell morphology and adhesion following alterations in the TTL-expression profile in Figure 8. A reduction in TTL-expression in the initial phase of epithelial monolayer formation goes along with an increase in detyr-tubulin enriched microtubules, integrin-integration into the basal membrane and stability of cell adhesion foci. The basal membrane is enlarged and cell attachment is elevated. On the other hand, enhanced TTL-expression diminishes the amount of detyr-tubulin enriched microtubules, focal adhesion foci, and adhesion capacity of the epithelial cell. Consequently, the basal membrane has a smaller diameter and the cell’s height increases to form a columnar cell shape. Altogether, these findings shed new light on the roles that tubulin-detyrosination plays in epithelial cell architecture and also in epithelial organization *per se*.

## Data Availability Statement

The original contributions presented in the study are included in the article/Supplementary Material, further inquiries can be directed to the corresponding author/s.

## Ethics Statement

The studies involving human participants were reviewed and approved by the Ethics Committee Faculty of Medicine University of Marburg. The patients/participants provided their written informed consent to participate in this study.

## Author Contributions

MM, KR, FH, and NK performed material preparation, data collection, and analysis. FH and TW performed the organoid analysis. RJ wrote the first draft of the manuscript. All authors contributed to the study conception and design, commented on previous versions of the manuscript and, read and approved the final manuscript.

## Conflict of Interest

The authors declare that the research was conducted in the absence of any commercial or financial relationships that could be construed as a potential conflict of interest.

## References

[B1] Aillaud C. Bosc C. Peris L. Bosson A. Heemeryck P. Van Dijk J. (2017). Vasohibins/SVBP are tubulin carboxypeptidases (TCPs) that regulate neuron differentiation. *Science* 358 1448–1453. 10.1126/science.aao4165 29146868

[B2] Balzer E. M. Tong Z. Paul C. D. Hung W. C. Stroka K. M. Boggs A. E. (2012). Physical confinement alters tumor cell adhesion and migration phenotypes. *FASEB J.* 26 4045–4056. 10.1096/fj.12-211441 22707566 PMC3448771

[B3] Bance B. Seetharaman S. Leduc C. Boeda B. Etienne-Manneville S. (2019). Microtubule acetylation but not detyrosination promotes focal adhesion dynamics and astrocyte migration. *J. Cell Sci.* 132:jcs225805. 10.1242/jcs.225805 30858195

[B4] Bershadsky A. Chausovsky A. Becker E. Lyubimova A. Geiger B. (1996). Involvement of microtubules in the control of adhesion-dependent signal transduction. *Current Biol.* 6 1279–1289. 10.1016/s0960-9822(02)70714-8 8939572

[B5] Borten M. A. Bajikar S. S. Sasaki N. Clevers H. Janes K. A. (2018). Automated brightfield morphometry of 3D organoid populations by OrganoSeg. *Sci. Rep.* 8:5319. 10.1038/s41598-017-18815-8 PMC5871765 29593296

[B6] Bouchet B. P. Gough R. E. Ammon Y. C. van de Willige D. Post H. Jacquemet G. (2016). Talin-KANK1 interaction controls the recruitment of cortical microtubule stabilizing complexes to focal adhesions. *Elife* 5:e18124. 10.7554/eLife.18124 PMC4995097 27410476

[B7] Bretscher M. S. Aguado-Velasco C. (1998). Membrane traffic during cell locomotion. *Curr. Opin. Cell Biol.* 10 537–541. 10.1016/s0955-0674(98)80070-7 9719876

[B8] Dorup J. Maunsbach A. B. (1997). Three-dimensional organization and segmental ultrastructure of rat proximal tubules. *Exp. Nephrol.* 5 305–317. 9259185

[B9] Erck C. Peris L. Andrieux A. Meissirel C. Gruber A. D. Vernet M. (2005). A vital role of tubulin-tyrosine-ligase for neuronal organization. *Proc.Natl.Acad.Sci.U.S.A* 102 7853–7858. 10.1073/pnas.0409626102 15899979 PMC1129054

[B10] Ezratty E. J. Bertaux C. Marcantonio E. E. Gundersen G. G. (2009). Clathrin mediates integrin endocytosis for focal adhesion disassembly in migrating cells. *J. Cell Biol.* 187 733–747. 10.1083/jcb.200904054 19951918 PMC2806590

[B11] Fellmann C. Hoffmann T. Sridhar V. Hopfgartner B. Muhar M. Roth M. (2013). An optimized microRNA backbone for effective single-copy RNAi. *Cell Rep.* 5 1704–1713. 10.1016/j.celrep.2013.11.020 24332856

[B12] Fourriere L. Kasri A. Gareil N. Bardin S. Bousquet H. Pereira D. (2019). RAB6 and microtubules restrict protein secretion to focal adhesions. *J. Cell Biol.* 218 2215–2231. 10.1083/jcb.201805002 31142554 PMC6605799

[B13] Gundersen G. G. Bulinski J. C. (1988). Selective stabilization of microtubules oriented toward the direction of cell migration. *Proc.Natl.Acad.Sci.U.S.A* 85 5946–5950. 10.1073/pnas.85.16.5946 3413068 PMC281882

[B14] Horton E. R. Byron A. Askari J. A. Ng D. H. J. Millon-Fremillon A. Robertson J. (2015). Definition of a consensus integrin adhesome and its dynamics during adhesion complex assembly and disassembly. *Nat. Cell Biol.* 17 1577–1587. 10.1038/ncb3257 26479319 PMC4663675

[B15] Iida-Norita R. Kawamura M. Suzuki Y. Hamada S. Masamune A. Furukawa T. (2019). Vasohibin-2 plays an essential role in metastasis of pancreatic ductal adenocarcinoma. *Cancer Sci.* 110 2296–2308. 10.1111/cas.14041 31074083 PMC6609860

[B16] Janke C. Magiera M. M. (2020). The tubulin code and its role in controlling microtubule properties and functions. *Nat. Rev. Mole. Cell Biol.* 21 307–326. 10.1038/s41580-020-0214-3 32107477

[B17] Kato C. Miyazaki K. Nakagawa A. Ohira M. Nakamura Y. Ozaki T. (2004). Low expression of human tubulin tyrosine ligase and suppressed tubulin tyrosination/detyrosination cycle are associated with impaired neuronal differentiation in neuroblastomas with poor prognosis. *Int. J.Cancer* 112 365–375. 10.1002/ijc.20431 15382060

[B18] Kaverina I. Rottner K. Small J. V. (1998). Targeting, capture, and stabilization of microtubules at early focal adhesions. *J. Cell Biol.* 142 181–190. 10.1083/jcb.142.1.181 9660872 PMC2133026

[B19] Keefe D. M. Brealey J. Goland G. J. Cummins A. G. (2000). Chemotherapy for cancer causes apoptosis that precedes hypoplasia in crypts of the small intestine in humans. *Gut* 47 632–637. 10.1136/gut.47.5.632 11034578 PMC1728102

[B20] Kim D. H. Wirtz D. (2013). Focal adhesion size uniquely predicts cell migration. *FASEB J.* 27 1351–1361. 10.1096/fj.12-220160 23254340 PMC3606534

[B21] Lafanechere L. Courtay-Cahen C. Kawakami T. Jacrot M. Rudiger M. Wehland J. (1998). Suppression of tubulin tyrosine ligase during tumor growth. *J. Cell Sci.* 111 171–181. 9405300 10.1242/jcs.111.2.171

[B22] Mialhe A. Lafanechere L. Treilleux I. Peloux N. Dumontet C. Bremond A. (2001). Tubulin detyrosination is a frequent occurrence in breast cancers of poor prognosis. *Cancer Res.* 61 5024–5027. 11431336

[B23] Morioka Y. Monypenny J. Matsuzaki T. Shi S. Alexander D. B. Kitayama H. (2009). The membrane-anchored metalloproteinase regulator RECK stabilizes focal adhesions and anterior-posterior polarity in fibroblasts. *Oncogene* 28 1454–1464. 10.1038/onc.2008.486 19169281

[B24] Nagae S. Meng W. Takeichi M. (2013). Non-centrosomal microtubules regulate F-actin organization through the suppression of GEF-H1 activity. *Genes Cells* 18 387–396. 10.1111/gtc.12044 23432781

[B25] Nieuwenhuis J. Adamopoulos A. Bleijerveld O. B. Mazouzi A. Stickel E. Celie P. (2017). Vasohibins encode tubulin detyrosinating activity. *Science* 358 1453–1456. 10.1126/science.aao5676 29146869

[B26] Palazzo A. F. Cook T. A. Alberts A. S. Gundersen G. G. (2001). mDia mediates Rho-regulated formation and orientation of stable microtubules. *Nat. Cell Biol.* 3 723–729. 10.1038/35087035 11483957

[B27] Palazzo A. F. Eng C. H. Schlaepfer D. D. Marcantonio E. E. Gundersen G. G. (2004). Localized stabilization of microtubules by integrin- and FAK-facilitated Rho signaling. *Science* 303 836–839. 10.1126/science.1091325 14764879

[B28] Peris L. Thery M. Faure J. Saoudi Y. Lafanechere L. Chilton J. K. (2006). Tubulin tyrosination is a major factor affecting the recruitment of CAP-Gly proteins at microtubule plus ends. *J. Cell Biol.* 174 839–849. 10.1083/jcb.200512058 16954346 PMC2064338

[B29] Prota A. E. Magiera M. M. Kuijpers M. Bargsten K. Frey D. Wieser M. (2013). Structural basis of tubulin tyrosination by tubulin tyrosine ligase. *J. Cell Biol.* 200 259–270. 10.1083/jcb.201211017 23358242 PMC3563685

[B30] Quinones G. B. Danowski B. A. Devaraj A. Singh V. Ligon L. A. (2011). The Post-Translational Modification of Tubulin Undergoes a Switch from Detyrosination to Acetylation as Epithelial Cells Become Polarized. *Mol. Biol. Cell* 2 1045–1057. 10.1091/mbc.e10-06-0519 21307336 PMC3069008

[B31] Rafiq N. B. M. Nishimura Y. Plotnikov S. V. Thiagarajan V. Zhang Z. Shi S. (2019). A mechano-signalling network linking microtubules, myosin IIA filaments and integrin-based adhesions. *Nat. Mater* 18 638–649. 10.1038/s41563-019-0371-y 31114072

[B32] Ran F. A. Hsu P. D. Wright J. Agarwala V. Scott D. A. Zhang F. (2013). Genome engineering using the CRISPR-Cas9 system. *Nat. Protoc.* 8 2281–2308. 10.1038/nprot.2013.143 24157548 PMC3969860

[B33] Raybin D. Flavin M. (1975). An enzyme tyrosylating alpha-tubulin and its role in microtubule assembly. *Biochem. Biophys. Res. Commun.* 65 1088–1095. 10.1016/s0006-291x(75)80497-9 1156416

[B34] Ridley A. J. Schwartz M. A. Burridge K. Firtel R. A. Ginsberg M. H. Borisy G. (2003). Cell migration: integrating signals from front to back. *Science* 302 1704–1709. 10.1126/science.1092053 14657486

[B35] Robison P. Caporizzo M. A. Ahmadzadeh H. Bogush A. I. Chen C. Y. Margulies K. B. (2016). Detyrosinated microtubules buckle and bear load in contracting cardiomyocytes. *Science* 352:aaf0659. 10.1126/science.aaf0659 27102488 PMC5441927

[B36] Roll-Mecak A. (2020). The Tubulin Code in Microtubule Dynamics and Information Encoding. *Dev. Cell* 54 7–20. 10.1016/j.devcel.2020.06.008 32634400 PMC11042690

[B37] Rong L. Bian Y. Liu S. Liu X. Li X. Liu H. (2017). Identifying tumor promoting genomic alterations in tumor-associated fibroblasts via retrovirus-insertional mutagenesis. *Oncotarget* 8 97231–97245. 10.18632/oncotarget.21881 29228606 PMC5722558

[B38] Soucek K. Kamaid A. Phung A. D. Kubala L. Bulinski J. C. Harper R. W. (2006). Normal and prostate cancer cells display distinct molecular profiles of alpha-tubulin posttranslational modifications. *Prostate* 66 954–965. 10.1002/pros.20416 16541425

[B39] Stehbens S. Wittmann T. (2012). Targeting and transport: how microtubules control focal adhesion dynamics. *J. Cell Biol.* 198 481–489. 10.1083/jcb.201206050 22908306 PMC3514042

[B40] Stubb A. Guzman C. Narva E. Aaron J. Chew T. L. Saari M. (2019). Superresolution architecture of cornerstone focal adhesions in human pluripotent stem cells. *Nat. Commun.* 10:4756. 10.1038/s41467-019-12611-w PMC6802214 31628312

[B41] Sumigray K. D. Terwilliger M. Lechler T. (2018). Morphogenesis and Compartmentalization of the Intestinal Crypt. *Dev. Cell* 18:e5. 10.1016/j.devcel.2018.03.024 PMC5987226 29689194

[B42] Tabbaa M. G. Axon A. T. R. Dixon M. F. (1994). Enterocyte dimensions in patients with abnormal intestinal permeability. *Eur. J. Gastroenterol. Hepatol.* 6 607–610. 10.1097/00042737-199407000-00008

[B43] Wang R. Yu R. Zhu C. Lin H. Y. Lu X. Wang H. (2019). Tubulin detyrosination promotes human trophoblast syncytium formation. *J. Mol. Cell Biol.* 11 967–978. 10.1093/jmcb/mjz084 31408157 PMC6927241

[B44] Whipple R. A. Cheung A. M. Martin S. S. (2007). Detyrosinated microtubule protrusions in suspended mammary epithelial cells promote reattachment. *Exp. Cell Res.* 313 1326–1336. 10.1016/j.yexcr.2007.02.001 17359970 PMC3132414

[B45] Williams J. M. Duckworth C. A. Burkitt M. D. Watson A. J. Campbell B. J. Pritchard D. M. (2015). Epithelial cell shedding and barrier function: a matter of life and death at the small intestinal villus tip. *Vet. Pathol.* 52 445–455. 10.1177/0300985814559404 25428410 PMC4441880

[B46] Wittmann T. Bokoch G. M. Waterman-Storer C. M. (2003). Regulation of leading edge microtubule and actin dynamics downstream of Rac1. *J. Cell Biol.* 161 845–851. 10.1083/jcb.200303082 12796474 PMC2172968

[B47] Zhai X. Y. Thomsen J. S. Birn H. Kristoffersen I. B. Andreasen A. Christensen E. I. (2006). Three-dimensional reconstruction of the mouse nephron. *J. Am. Soc. Nephrol.* 17 77–88. 10.1681/asn.2005080796 16319188

[B48] Zhang X. Tee Y. H. Heng J. K. Zhu Y. Hu X. Margadant F. (2010). Kinectin-mediated endoplasmic reticulum dynamics supports focal adhesion growth in the cellular lamella. *J. Cell Sci.* 123 3901–3912. 10.1242/jcs.069153 20980389 PMC3705938

[B49] Zink S. Grosse L. Freikamp A. Banfer S. Muksch F. Jacob R. (2012). Tubulin detyrosination promotes monolayer formation and apical trafficking in epithelial cells. *J. Cell Sci.* 125 5998–6008. 10.1242/jcs.109470 23097046

