# Supplementary material for: TTL-Expression Modulates Epithelial Morphogenesis
Source: Front Cell Dev Biol. 2021 Feb 5;9:635723. doi: 10.3389/fcell.2021.635723 (PMC7892909; doi:10.3389/fcell.2021.635723)
Supplement: Supplementary file 1 [file Data_Sheet_1.pdf]

Supplementary information

Figure S1

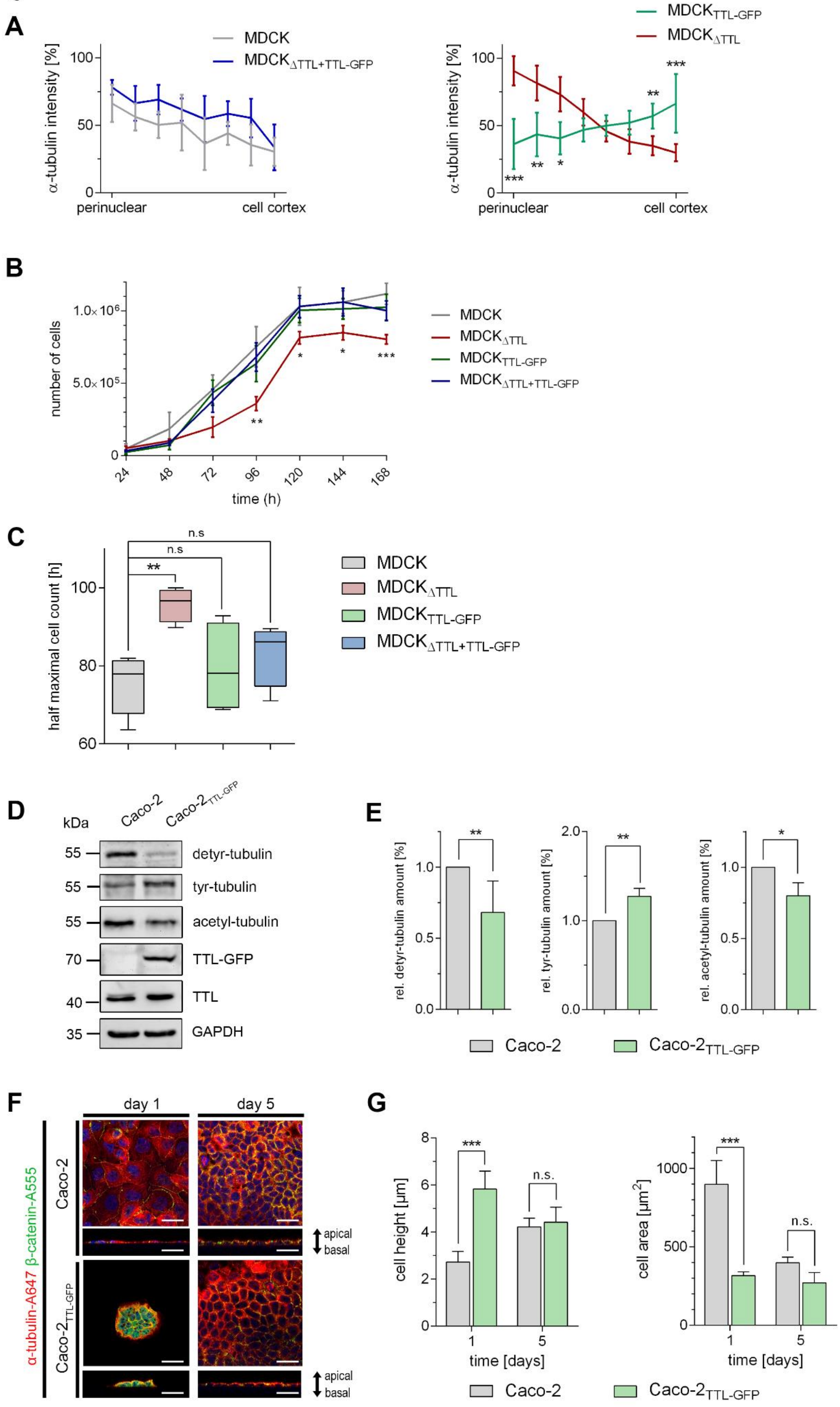

Figure S1: Proliferation of MDCK cell lines and characterization of Caco-2 and Caco-$2_{TTL\text{-}GFP}$ cells.

(A) One day after filter-seeding MDCK, $MDCK_{\Delta TTL}$, $MDCK_{TTL\text{-}GFP}$, and $MDCK_{\Delta TTL+TTL\text{-}GFP}$ cells were fixed and immunostained with pAb anti-β-catenin (Alexa Fluor 555) and mAb anti-α-tubulin (Alexa Fluor 647) (see also Fig. 1C). α-tubulin intensities were measured by line scan analysis from the perinuclear cell area to the cell cortex. 10 cells were analyzed from each experiment. Mean±s.d., n=3. Statistical significance was tested with two-way ANOVA and Bonferroni's post-test (*$P<0.05$; **$P<0.01$; ***$P<0.001$). (B, C) MDCK, $MDCK_{\Delta TTL}$, $MDCK_{TTL\text{-}GFP}$, and $MDCK_{\Delta TTL+TTL\text{-}GFP}$ cells were seeded on 12-well plates. After indicated time intervals cells were collected and the number of cells was counted. Three wells of every cell line were measured and the measurement was performed in triplicates. Mean±s.d. Statistical significance was tested with two-way ANOVA and Bonferroni's post-test (*$P<0.05$; ***$P<0.001$). (C) Comparison of half maximal cell count of MDCK, $MDCK_{\Delta TTL}$, $MDCK_{TTL\text{-}GFP}$, and $MDCK_{\Delta TTL+TTL\text{-}GFP}$ cells. Mean±s.d. Mean is shown as line. Statistical significance was tested using one-way ANOVA with Dunnet's comparison (n.s., not significant; **$P<0.01$). (D, E) Cellular levels of detyrosinated, tyrosinated and acetylated tubulin were assessed by Western blot analysis of cell lysates from polarized Caco-2 and Caco-$2_{TTL\text{-}GFP}$ cells. Protein concentrations of the lysates were determined and equal amounts were loaded on each lane of the SDS-PAGE. Relative protein expression was normalized to GAPDH levels. (E) Relative detyrosinated, tyrosinated and acetylated tubulin expression in each cell line as compared to Caco-2 cells. Quantities from Caco-2 cells were set as 1. n=3. Statistical significance was tested using Student's t-test (n.s., not significant; *$P<0.05$; **$P<0.01$). (F, G) At indicated time intervals after filter-seeding Caco-2 cells were fixed and immunostained with pAb anti-β-catenin (Alexa Fluor 555) and mAb anti-α-tubulin (Alexa Fluor 647). Xy-scans and xz-scans are depicted for each time interval and each cell line. Nuclei are indicated in blue; scale bars, 25 µm. (G) Quantification of cell height and cell area. Mean±s.d., n=3. Statistical significance was tested using Student's t-test (n.s., not significant; *** $P<0.001$).

Figure S2

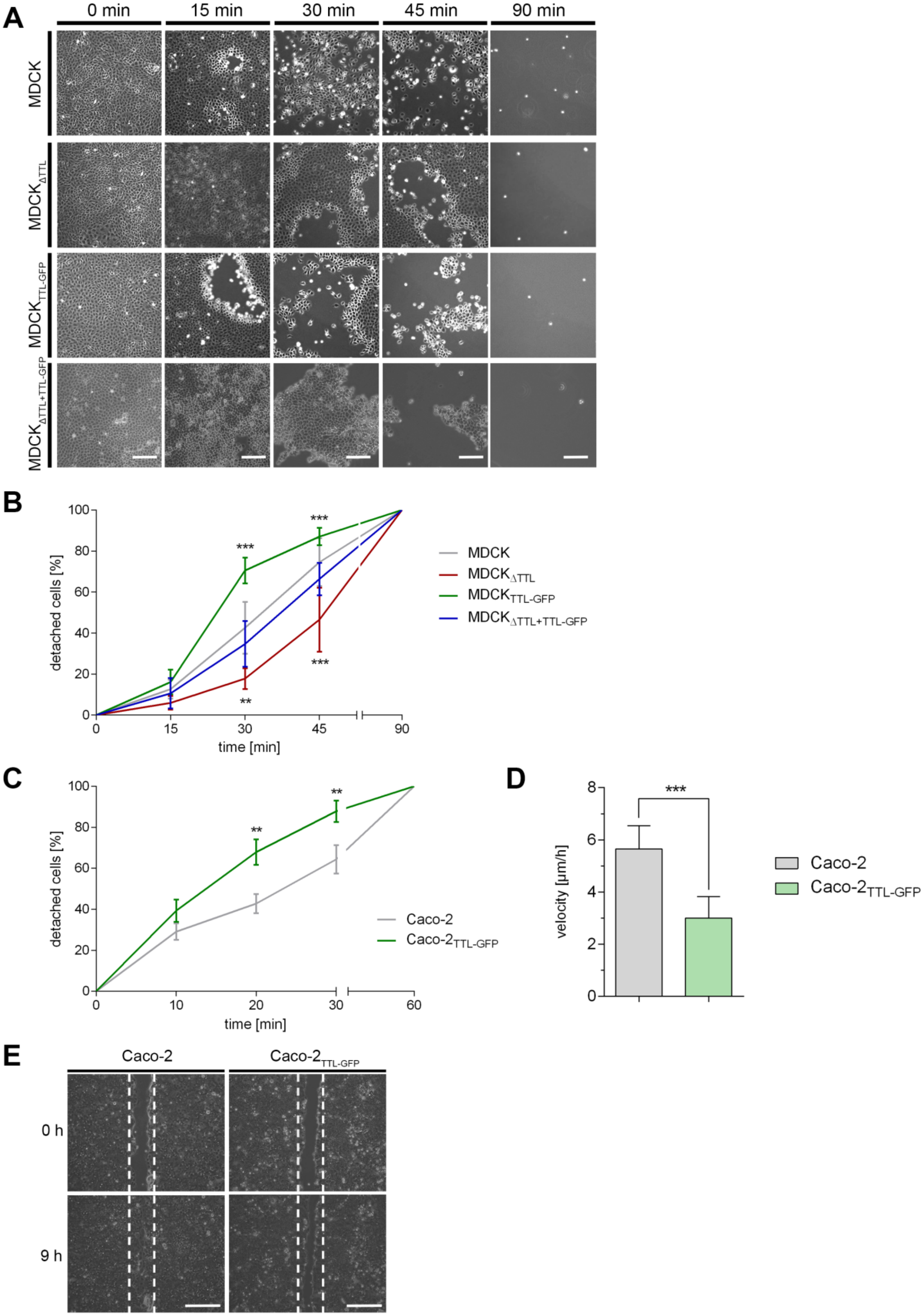
A
0 min
15 min
30 min
45 min
90 min
MDCK
MDCKΔTTL
MDCKTTL-GFP
MDCKΔTTL+TTL-GFP
B
detached cells [%]
time [min]
MDCK
MDCKΔTTL
MDCKTTL-GFP
MDCKΔTTL+TTL-GFP
C
detached cells [%]
time [min]
Caco-2
Caco-2TTL-GFP
D
velocity [µm/h]
Caco-2
Caco-2TTL-GFP
E
Caco-2
Caco-2TTL-GFP
0 h
9 h

Figure S2: Tubulin detyrosination and cell adhesion/ migration of MDCK and Caco-2 cells.

(A, B) A trypsin-based cell de-adhesion assay was performed to measure the strength of cell attachment to collagen type I coated culture dishes. Confluent cell layers of MDCK, $MDCK_{\Delta TTL}$, $MDCK_{TTL\text{-}GFP}$, and $MDCK_{\Delta TTL+TTL\text{-}GFP}$ cells were washed with PBS and then incubated with warm trypsin/EDTA (0.05%/0.02%) for indicated time intervals. Scale bar: 100 µm. (B) Quantitative results of the de-adhesion assay showing percentage of detached cells. Mean±s.d., n=3. Statistical significance was tested with two-way ANOVA and Bonferroni's post-test (n.s., not significant; *P<0.05; **P<0.01; *** P<0.001). (C) A trypsin-based cell de-adhesion assay was performed to measure the strength of cell attachment. Confluent cell layers of Caco-2 and Caco-$2_{TTL\text{-}GFP}$ cells were washed with PBS and then incubated with warm trypsin/EDTA (0.05%/0.02%) for indicated time intervals. Quantitative results of the de-adhesion assay showing percentage of detached cells are indicated. Mean±s.d., n=4. Statistical significance was tested with two-way ANOVA and Bonferroni's post-test (n.s., not significant; **P*<0.05; ***P*<0.01; ****P*<0.001). (D, E) Confluent monolayers of Caco-2 and Caco-$2_{TTL\text{-}GFP}$ cells were scratch wounded to analyze migration. Cells were recorded at 0, 3, 6 (3 and 6 not shown) and 9 h post-scratching. Mean cell migration velocity was calculated. Mean±s.d., n=6. Statistical significance was tested with Student's *t*-test (****P*<0.001). (E) Images recorded immediately after (0 h) or 9 h post scratching are depicted. White dotted lines indicate the wound borders at the beginning of the assay. Scale bars: 100 µm.

Figure S3

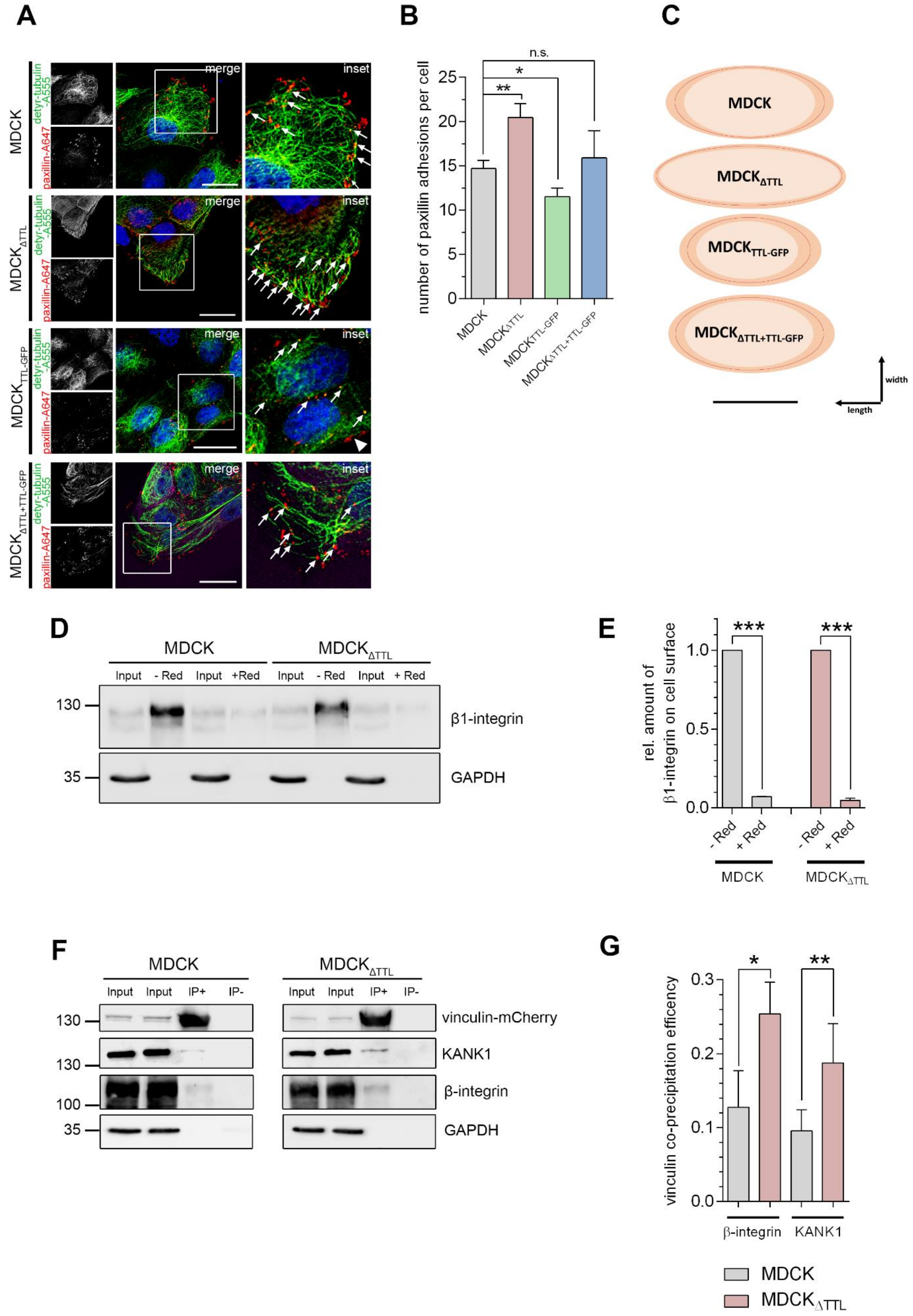
A
B
C
MDCK
MDCK$_{\Delta TTL}$
MDCK$_{TTL\text{-}GFP}$
MDCK$_{\Delta TTL+TTL\text{-}GFP}$
detyr-tubulin -A555
paxillin-A647
merge
inset
n.s.
number of paxillin adhesions per cell
width
length
D
β1-integrin
GAPDH
Input
- Red
+Red
E
rel. amount of β1-integrin on cell surface
F
vinculin-mCherry
KANK1
β-integrin
IP+
IP-
G
vinculin co-precipitation efficency

Figure S3: Number/ size of paxillin-positive focal adhesions and interaction between vinculin- or paxillin-mCherry with detyr-, tyr- or acetylated tubulin.

(A-C) Subconfluent MDCK, $MDCK_{\Delta TTL}$, $MDCK_{TTL\text{-}GFP}$ and $MDCK_{\Delta TTL+TTL\text{-}GFP}$ cells were immunostained with pAb anti-detyr-tubulin (Alexa Fluor 555) and mAb anti-paxillin (Alexa Fluor 647). Arrows indicate colocalization of detyrosinated microtubule and paxillin. Scale bar: 25 µm. (B) Quantification of paxillin-positive focal adhesions per cell. Mean±s.d., 8 – 10 cells per experiment, n=3 independent experiments. Statistical significance was tested with Student`s unpaired t-test (n.s., not significant; $^{**}P<0.01$). (C) Schematic diagram showing the average size and shape from paxillin-positive focal adhesions in MDCK, $MDCK_{\Delta TTL}$, $MDCK_{TTL\text{-}GFP}$ and $MDCK_{\Delta TTL+TTL\text{-}GFP}$ cells. Shape of focal adhesions is shown as ovals with best fit around length and width. Average sizes are indicated by red lines, s.d. is depicted in orange. 15 – 20 focal adhesions were measured per experiment. n=3 independent experiments. Scale bar: 1 µm. (D, E) In control experiments for Figure 7A, B the efficiency of β1-integrin-biotinylation and biotin-removal by reduced glutathione was determined. Membrane proteins of MDCK and $MDCK_{\Delta TTL}$ cells were biotinylated with NHS-SS-biotin. Internalization was blocked at 4 °C and biotin label was removed (+Red) or not removed (-Red) by reduced glutathione. Cells were lysed, biotinylated proteins were precipitated with neutravidin-beads and precipitates were analyzed by immunoblot against β1-integrin and GAPDH. (E) Quantification of (D). Mean±s.d., n=3. Statistical significance was tested with Students t-test. Note efficient removal of biotin-label from β1-integrin if internalization was blocked. (F) Co-precipitation of β1-integrin or KANK1 and vinculin-mCherry. MDCK cells transfected with vinculin-mCherry were lysed 36 h post-transfection. Cell lysates were incubated with RFP-Trap beads or blocked agarose beads (negative control). Western Blots were incubated with pAb anti-CD29 (β1-integrin), pAb anti-KANK1 and mAb anti-vinculin. (G) Quantification of the co-precipitation efficiency of β1-integrin and KANK1 from three independent experiments. The efficiency was normalized by the total quantities of each polypeptide in the input. Statistical significance was tested with Student`s unpaired t-test ($^{*}P<0.05$).

Figure S4

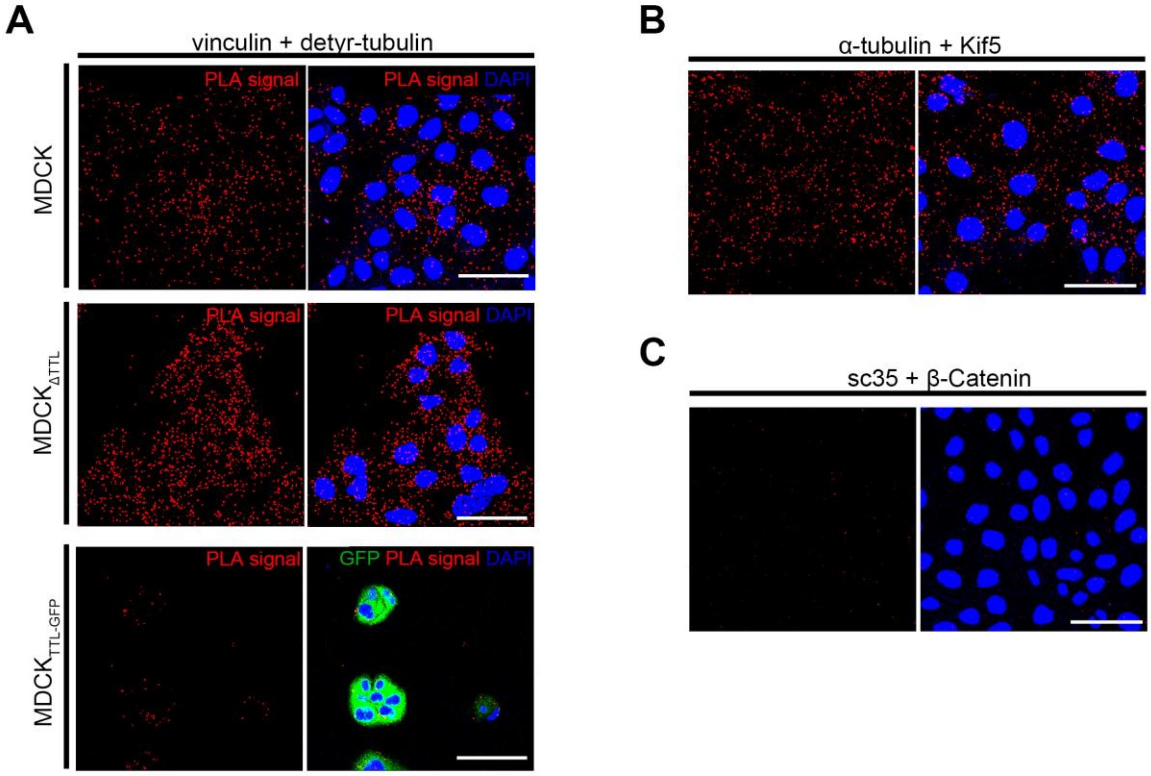

Figure S4: Correlation between tubulin detyrosination and the number/ size of vinculin-positive focal adhesions.

(A) Non-polarized MDCK, MDCK$_{\Delta TTL}$ and MDCK$_{TTL-GFP}$ cells were incubated with antibodies against vinculin and detyr-tubulin. Each red signal indicates close proximity between vinculin and detyr-tubulin. Nuclei are indicated in blue. Images are representative for 3 independent experiments. Scale bars: 50 µm. PLA analysis of α-tubulin/ kinesin motor Kif5 as a positive (B) and sc35/ β-catenin as a negative control (C). Nuclei are depicted in blue. Scale bars: 50 µm.

Figure S5

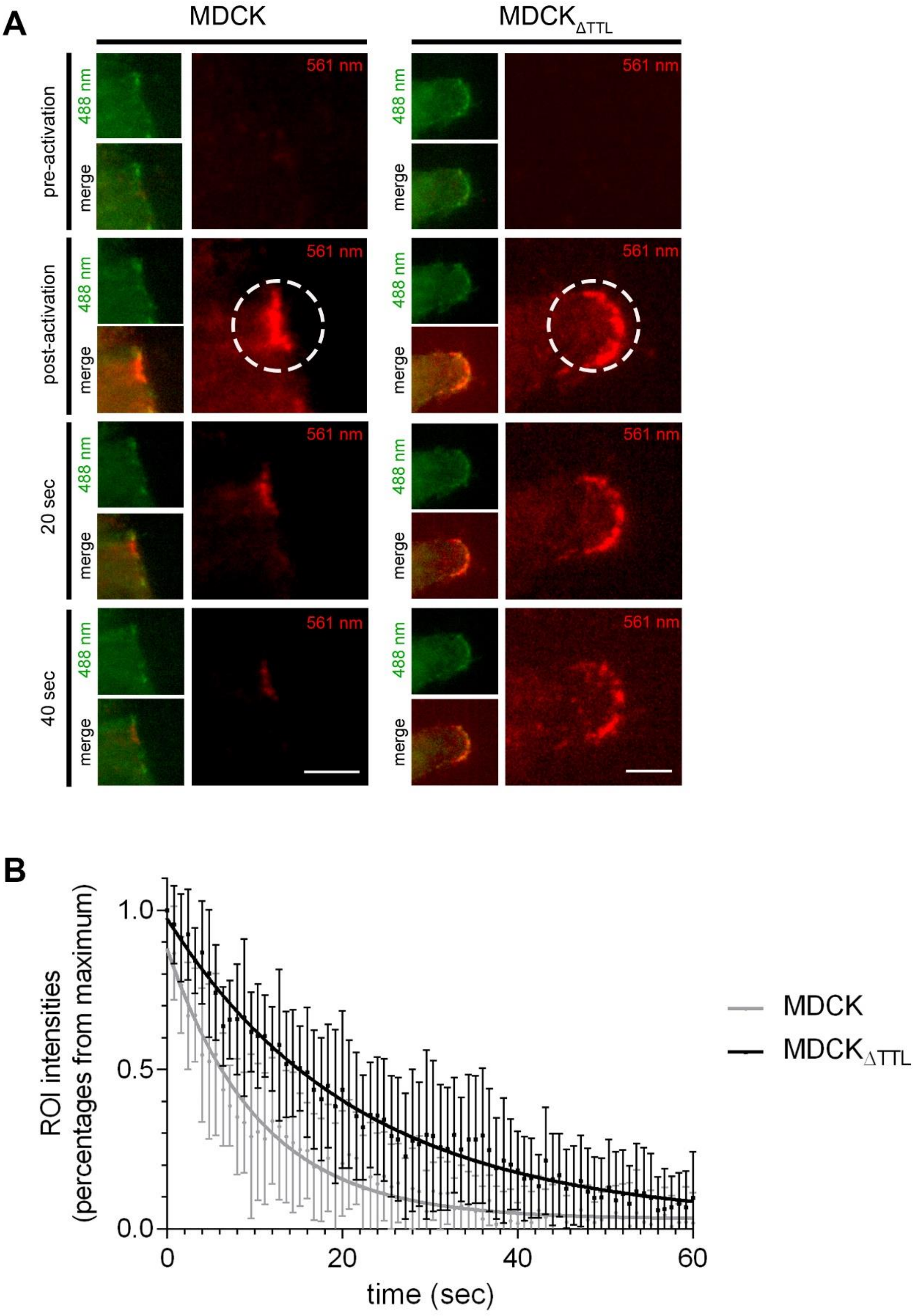
A
MDCK
MDCK$_{\Delta TTL}$
pre-activation
post-activation
20 sec
40 sec
488 nm
merge
561 nm
B
ROI intensities (percentages from maximum)
1.0
0.5
0.0
0
20
40
60
time (sec)
MDCK
MDCK$_{\Delta TTL}$

Figure S5: Tracking vinculin dynamics by photoconversion of vinculin-mEOS2.

(A, B) MDCK and $MDCK_{\Delta TTL}$ cells were incubated on glass bottom WillCo dishes under a humidified atmosphere of 5% CO2 in air. Images were recorded from cells by 475 nm excitation/ 531 nm emission (green) or 575 nm excitation/ 589 nm emission (red) before and after photoconversion of indicated cell areas (regions of interest, dotted lines). For photoconversion of vinculin-mEOS2 focal adhesions in the cell periphery were illuminated for 5 sec at 405 nm. Scale bars: 5 µm. (B) Experiments were quantified with the Leica LAS AF software to plot the dissociation kinetics of vinculin-mEOS2. n=3.
